# The effect of lyophilised oral faecal microbial transplantation on functional outcomes in dogs with diabetes mellitus

**DOI:** 10.1111/jsap.13865

**Published:** 2025-04-15

**Authors:** R. Brown, P. Barko, J. D. J. Ruiz Romero, D. A. Williams, A. Gochenauer, J. Nguyen‐Edquilang, J. S. Suchodolski, R. Pilla, H. Ganz, N. Lopez‐Villalobos, A. Gal

**Affiliations:** ^1^ Department of Veterinary Clinical Medicine University of Illinois at Urbana‐Champaign Urbana Illinois USA; ^2^ Gastrointestinal Laboratory, Department of Small Animal Clinical Sciences Texas A&M University College Station Texas USA; ^3^ AnimalBiome Oakland California USA; ^4^ School of Agriculture and Environment Massey University Palmerston North New Zealand

## Abstract

**Objectives:**

We aimed to determine if oral faecal microbiota transplantation improves indices of glycaemic control, changes the faecal dysbiosis indices, alters faecal short‐chain fatty acid and bile acid profiles and increases serum glucagon‐like‐peptide 1 concentrations in diabetic dogs.

**Materials and Methods:**

In this prospective randomised, placebo‐controlled, double‐blinded pilot study, we recruited nine diabetic dogs (five faecal microbiota transplantation and four placebo) and nine healthy controls.

**Results:**

Compared to healthy dogs, diabetic dogs had altered faecal short‐chain fatty acid and bile acid profiles. In the first 30 days, the faecal microbiota transplantation group had a more rapid decline in interstitial glucose; however, the mean interstitial glucose of the faecal microbiota transplantation recipients did not differ from the placebo recipients at the end of the study. Compared with placebo, faecal microbiota transplantation recipients had a decreased 24‐hour water intake at day 60 and increased faecal abundance of *Faecalibacterium*.

**Clinical Significance:**

This study provides a proof of concept for faecal microbiota transplantation in canine diabetes, and its data could inform the design of future large‐scale studies. Further investigation is required to determine whether faecal microbiota transplantation would have any role as an adjunctive therapy in canine diabetes and to elucidate the mechanisms by which faecal microbiota transplantation may provide a beneficial clinical effect in canine diabetes.

## INTRODUCTION

Diabetes mellitus (DM) is one of the most common endocrine diseases in dogs, with a prevalence ranging between 0.15% and 1.33% (Davison et al., 2005; Fracassi et al., 2004; Guptill et al., 2003; Mattin et al., 2014; Wiles et al., 2017). In most diabetic dogs, beta‐cells are lost, leading to a deficiency in insulin production and an absolute requirement for exogenous insulin. Previous studies indicated that dogs with diabetes may have changes in the composition of gut microbial communities, including bacteria that are associated with the fermentation of short‐chain fatty acids (SCFAs) (Jergens et al., 2019; Laia et al., 2022). If changes in the composition of gut microbial communities (i.e., gut dysbiosis) are mechanistically linked to canine DM, then restoring a state of eubiosis may possibly have a positive effect on the management of canine DM.

Evidence from diabetic humans and diabetic animal models suggest that the intestine and gut dysbiosis may intersect with DM in part through the “leaky gut syndrome” (Iatcu et al., 2021; Tanase et al., 2020; Wang et al., 2019; Zhang et al., 2020; Zhou et al., 2022). Gut leakiness leads to a state of chronic inflammation resulting in reduced insulin sensitivity (Tanase et al., 2020). Short‐chain fatty acids (SCFA) that are derived from gut microbiome (GM) metabolism contribute to gut leakiness. SCFAs signalling through the G‐protein‐coupled receptors 43 and 41 (GPR43/FFA2 and GPR41/FFA3) also induce the secretion of glucagon‐like‐peptide 2 (GLP‐2) which is important for the integrity of epithelial tight junctions (Tanase et al., 2020; Zhou et al., 2022). Independently, hyperglycaemia also has a direct negative effect on the integrity of the gut epithelial tight junctions (Thaiss et al., 2018). The incretin hormone glucagon‐like‐peptide 1 (GLP‐1) is upregulated by gut SCFAs and sensitises beta‐cells' insulin secretion (Tanase et al., 2020). Hence, severe downregulation of GLP‐1 in diabetes indirectly affects the gut barrier through hyperglycaemia. GLP‐1 also has glucose regulatory pleiotropic effects on cell signalling, metabolism and function beyond its direct effect on beta‐cells, which include increased satiety, decreased gastric emptying, inhibition of glucagon secretion, sensitisation of the liver to insulin (including exogenous insulins) and increased utilisation of glucose in skeletal muscles (Rowlands et al., 2018). Bile acids (BAs) modulate gut leakiness through epidermal growth factor (EGF) receptor autophosphorylation, occludin dephosphorylation and rearrangement at the tight junction level (Raimondi et al., 2008). Secondary BAs (SBAs) that are metabolised by gut microbes signal through the G‐protein‐coupled bile acid receptor 1 (TGR5) and the BA farnesoid X receptor (FXR) (Ferrell & Chiang, 2019). SBAs induce GLP‐1 secretion and also affects lipid and glucose metabolism via regulation of hepatic very low‐density lipoproteins (VLDL) export, triglyceride oxidation and gluconeogenesis (Ferrell & Chiang, 2019). Bacterial‐derived metabolites that have been associated with the “leaky gut syndrome” include indole metabolites of tryptophan (IMT), trimethylamine (TMA) and branched‐chain amino acids (BCAAs). These metabolites can negatively affect the sensitivity to insulin (Ma et al., 2017; Scott et al., 2020; Seldin et al., 2016).

We hypothesised that faecal microbial transplantation (FMT) will improve indices of glycaemic control in FMT recipients compared to placebo control dogs and will increase the abundance of faecal *Fecalibacterium*, faecal SCFA concentrations and serum GLP‐1 concentrations. We therefore aimed to (1) determine if FMT will improve clinical outcomes of DM; (2) determine if changes in the faecal dysbiosis index (DI) and its components are associated with DM and FMT; and (3) measure the concentrations of faecal BA, SCFA and serum GLP‐1 in response to FMT.

## MATERIALS AND METHODS

**FIG 1:**
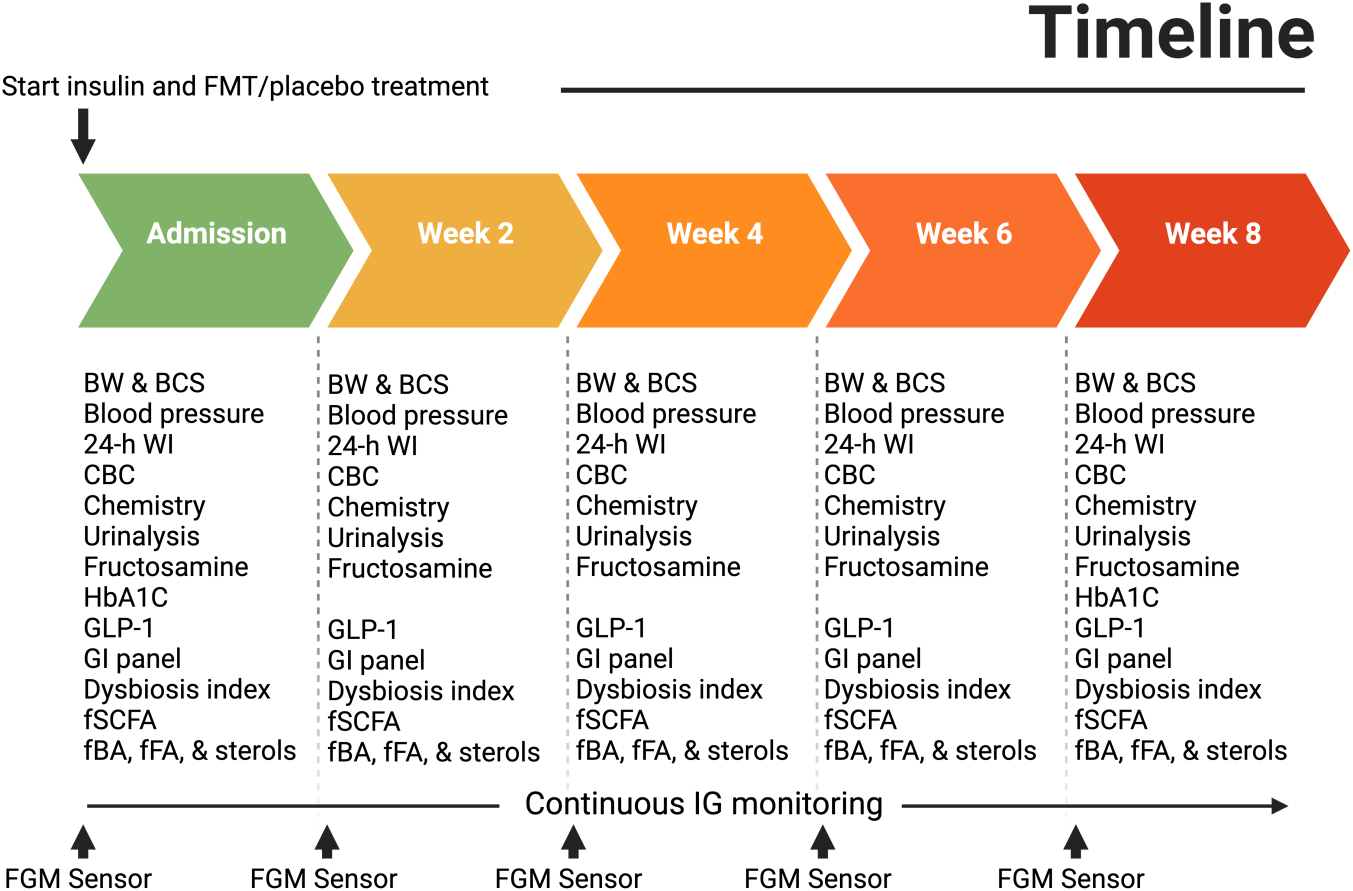
Study timeline and samples collections. BW Bodyweight, BCS Body condition score (1 to 9), WI 24‐Hour water intake, HbA1C Haemoglobin A1C, GLP‐1 Glucagon‐like peptide 1, GI Gastrointestinal, fSCFA Faecal short‐chain fatty acid, fBA Faecal bile acid, fFA Faecal fatty acid.

This report describes prospective randomised, placebo‐controlled, double‐blinded, clinical trial. We randomised the dogs in the study to placebo or FMT groups using an online randomisation tool (www.randomizer.org), and in this report, we describe the results of this pilot investigation. Between September 2021 and November 2021, we enrolled nine diabetic dogs from public and local referral clinics after receipt of each owner's written consent to participate in the study (demographic information of the dogs is present in Table 1). The inclusion criteria below aimed to try enrolling a relatively stable cohort of dogs with uncomplicated diabetes mellitus. The diabetic dogs were required to have been previously diagnosed with DM and treated with insulin for at least 14 days before enrolment. Additional enrolment criteria included a stable body weight (±5% change) for at least 14 days before enrolment, absence of profound clinical signs of DM (i.e. polydipsia, polyuria, polyphagia and weight loss) for at least 14 days prior to enrolment as determined by the primary investigator, an American College of Veterinary Internal Medicine (ACVIM) board‐certified internist, and no recent use of any steroids, antibiotics or probiotics within 14 days of enrolment. During the period of the study participants' diets were not changed (dietary nutritional analysis is present in Table S1). The dogs were assessed at admission and at 2, 4, 6 and 8 weeks by physical exam and a comprehensive health profile, where at each visit serum, urine and faeces were collected and frozen at −80°C for specific analyses at the end of the study (Fig 1). Throughout the 8 weeks of the study, the dogs continuously wore a flash glucose monitoring (FGM) device that measured the interstitial glucose (IG) concentration at 15 minutes epochs. The FGM was previously validated for use in diabetic dogs (Corradini et al., 2016). Owners were instructed to scan the sensors with the FGM every 6 hours to minimise data loss and to upload it daily to the University's account on the Libreview website (https://www.libreview.com). Sensors were changed at 2‐week intervals during the visits to the clinic, or earlier at home by the dogs' owners if the sensors malfunctioned. The mean (±SD) period of sensor coverage throughout the length of the study was 83% ± 14% (mean (±SD) 4820 ± 825 IG counts).

**Table 1:** Descriptive statistics (mean ± SD) and demographics of dogs in the study

	Healthy	DM	FMT	Placebo	Donor
Sex	CM (4), SF (4), IM (1)	CM (5), SF (4)	CM (3), SF (2)	CM (2), SF (2)	CM (3), SF (1)
Age (year)	6.4 ± 2.8^a^	10 ± 1.6^b^	9.7 ± 1.3	10.4 ± 2.1	5.8 ± 0.5^a^
Bodyweight (kg)	6.6 ± 3.5^a^	13.8 ± 8.7^b^	17.2 ± 9.4	9.6 ± 6.3	27.6 ± 7.2^c^
BCS	5.4 ± 0.9^a^	5.6 ± 1.1^a^	5.8 ± 1.1	5.3 ± 1.3	4.8 ± 0.5^a^
Breed	MBD (3), Miniature schnauzer (2), Miniature poodle, Pug, Pembroke Welsh Corgi, dachshund	MBD (5), Pug (2), Chihuahua, Australian cattle dog	MBD (3); Pug (2)	MBD (2), Chihuahua, Australian cattle dog	Australian cattle dog (1), Border collie/GSD mix (1), Pit bull mix (1), GSD (1)

BCS Body condition score (scale of 1 to 9), CM Castrated male, DM Diabetes mellitus (= FMT + placebo), FMT Faecal microbiota transplantation, GSD German shepherd, IM Intact male, SF Spayed female, MBD Mixed breed dog

Different superscript letters indicate a significant difference between groups (P < 0.05)

The University of Illinois Institutional Animal Care and Use Committee approved this study (Protocol#19235). Informed consent was obtained from all dog owners involved in the study. No participants are identifiable.

### 24‐hour water quantification

After joining the study and before starting insulin treatment, participants were directed to monitor the amount of water they placed in their dogs' bowls for a period of 24 hours. Throughout the study, these measurements were repeated every 2 weeks. If other dogs shared the household with study dogs, they were either denied access to water and offered water only during specific times and separately from the study dogs, or the study dogs were kept separate in a different room for 24 hours.

### Healthy control dogs

Archived (−80°C) serum and faecal samples from nine healthy dogs from Texas A&M University (Table 1) were chosen to serve as healthy control samples and were analysed side by side with the diabetic dog samples. These dogs were previously deemed healthy based on history, physical exam and a comprehensive health profile. Healthy controls were not included in the FMT clinical trial.

### Insulin treatment

**FIG 2:**
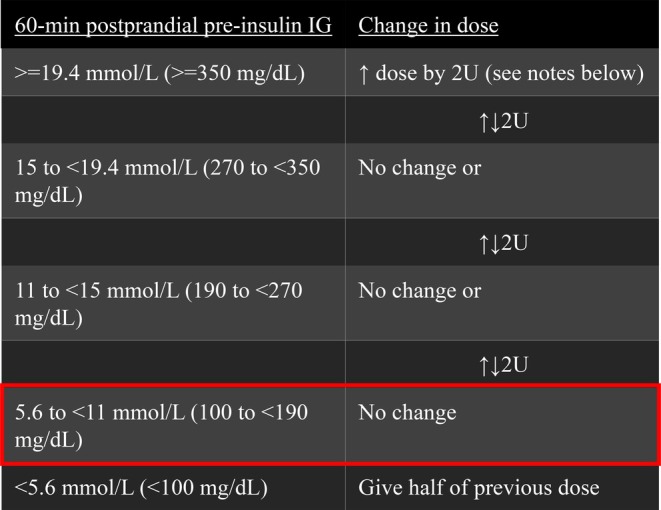
Sliding‐scale insulin dosing table for owners of diabetic dogs (BW >15 kg; for dogs with BW ≤15 kg, the change in insulin dose from one tier to the next is by 1 unit). The sliding‐scale insulin dosing approach aims to maintain interstitial glucose (IG) concentrations within a specific range through incremental insulin adjustments based on where the IG levels fall relative to a target tier. This approach has five tiers of IG concentrations. The goal is to maintain the IG concentration within a specific target tier that is highlighted in the figure in red. Dosing adjustment based on IG concentration: Within Target Tier: If the IG concentration falls within the desired tier, the insulin dose remains the same. Below Target Tier: If the IG concentration falls below the target tier, the insulin dose is continuously halved at each dosing interval. Moving from a higher tier to a lower tier: If IG concentration falls into a lower tier than the previous dosing interval's tier, the insulin dose is decreased by 2 units. The dose will not be adjusted in the following dosing interval if IG concentration falls into the same tier but will be increased by 2 units in the third dosing interval (or during additional subsequent intervals). Moving from a lower tier to a higher tier: If IG concentration falls into a higher tier than the previous dosing interval's tier, the insulin dose is increased by 2 units. The dose will not be adjusted in the following dosing interval if IG concentration falls into the same tier, but it will be increased by 2 units in the third dosing interval (or during additional subsequent intervals). Highest Tier: If the IG concentration falls into the highest tier, the insulin dose is increased continuously with each dosing interval.

At enrolment, all dogs were switched to Toujeo glargine insulin 300 U/mL (Toujeo SoloStar for dogs ≤15 kg and Toujeo Max SoloStar for dogs >15 kg) given every 12 hours at a starting dose approximating their insulin dose before enrolment. The owners of the diabetic dogs were instructed to follow a sliding‐scale insulin‐dosing table that based the insulin dose on the 60‐minute post‐prandial IG level (Fig 2). The table has five tiers of interstitial glucose ranges indicating an increase, decrease, or no change in insulin dose relative to the insulin dose of the previous dosing interval. The owners were granted access to an online Google Document in which they recorded the morning and evening insulin dose of each day. The rationale for this approach is to avoid bias and maintain consistency. By ensuring a uniform insulin dosage strategy across cases, we were attempting to minimise any potential influence that might arise from variations in insulin dosing on glycaemic control. Therefore, this approach allows for a more accurate assessment of the impact of FMT on glycaemic control without the confounding factor of inconsistent insulin dosing which could profoundly affect glycaemic control.

### Lyophilised faecal microbial transplantation

**FIG 3:**
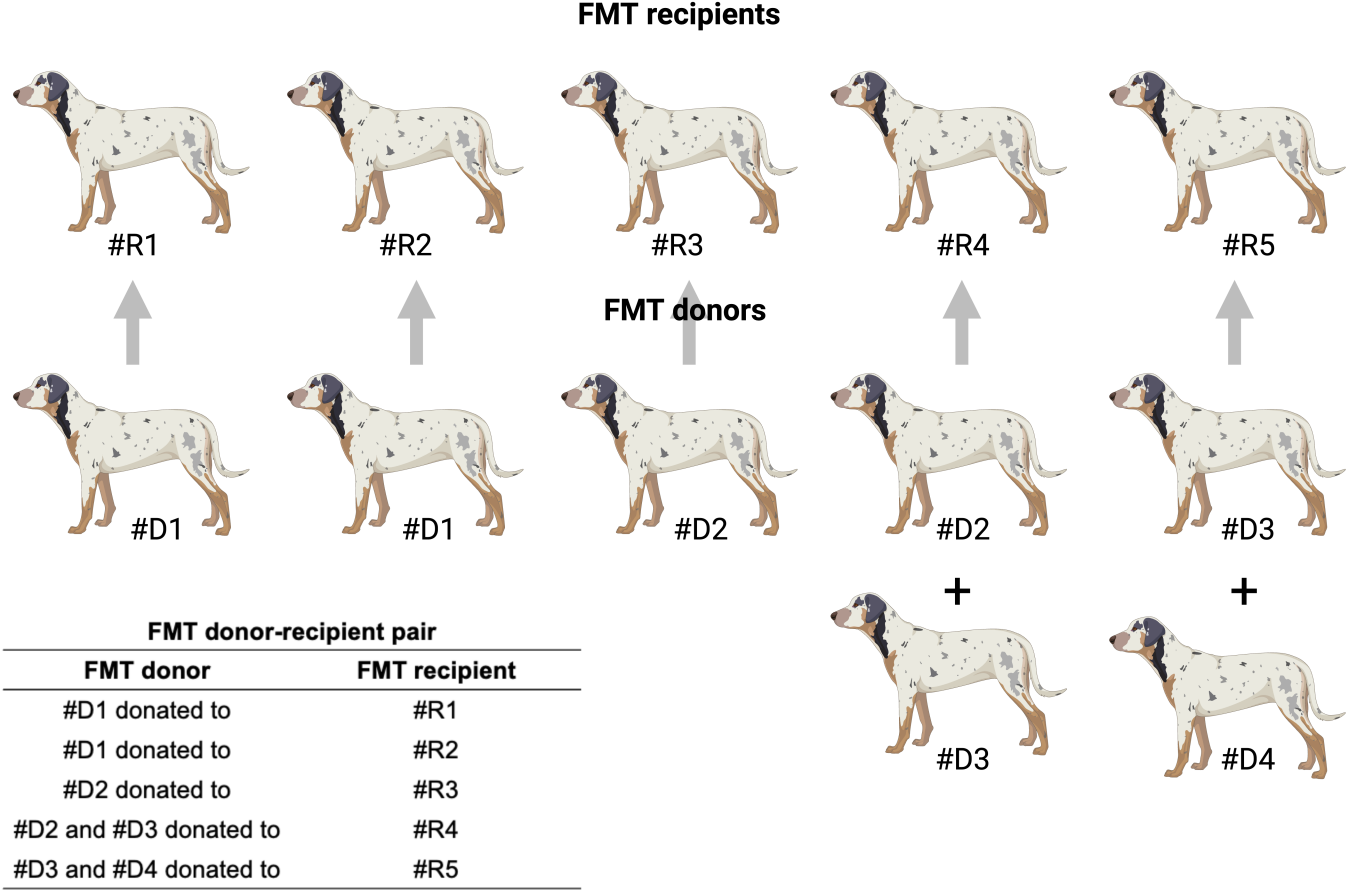
FMT donor‐recipient pairs. **#**D, FMT donor dog; #R, FMT recipient dog. Two FMT recipients each received a mixed FMT from two FMT donors.

The dogs in the FMT group received 1 g of lyophilised faeces (range 0.035 to 0.166 g/kg) daily from the owners in capsule form. The dose was extrapolated from a previous faecal transplantation study (Gal et al., 2021). The owners were instructed to record the daily administration of the lyophilised pill on an online Google Document shared with the PI to verify compliance. The lyophilised faecal capsules (size 0) were obtained from a commercial vendor. The demographic information of the vendor's donor dogs is present in Table 1. Placebo capsules (1 g cornstarch; range 0.041 to 0.217 g/kg) were also obtained from the same vendor and packaged in identical acid‐resistant size 0 capsules. To the best of our knowledge, cornstarch has not been established as a prebiotic in dogs at the dose that was administered in this study (Baioni et al., 2017). The randomisation table and identity of the capsules were only known to a veterinary pharmacist who oversaw the randomisation process and the distribution of placebo and FMT capsules to the owners of the patients at the time of enrolment and did not have any other roles in the study to maintain the double blinding process (Fig 3).

### Serum total GLP‐1

Blood was collected early in the morning prior to feeding, and serum was separated and kept frozen at −80°C until the time of analysis. 50 μL of thawed frozen serum was used to measure serum total GLP‐1 in one batch by GLP‐1 ELISA (Multi‐Species GLP‐1 Total ELISA kit, Millipore Corporation, Billerica, MA, USA) that has been previously used for dogs (Ionut et al., 2014) and in accordance with the manufacturer's instructions. According to the manufacturer, the assay's diagnostic range is 4.1 to 1000 pM, the assay's lowest limit of detection is 1.5 pM, and the intra‐assay coefficient of variation (CV%) is 5.5% (in canine plasma). The assays reactions were read on a microtitre plate reader (SpectraMax® ID3, Molecular Devices, LLC, San Jose, CA, USA) at absorbency of 450 and 590 nm.

### Serum gastrointestinal panel and C‐reactive protein, faecal DI, faecal fatty acids, faecal sterols and faecal unconjugated BAs

Serum trypsin‐like immunoreactivity (TLI), serum canine pancreatic lipase immunoreactivity (cPLI), serum CRP, serum folate, serum cobalamin and the faecal DI were measured at the Gastrointestinal Laboratory at Texas A&M as described previously for dogs (Chang et al., 2022; Covin et al., 2021). The DI is a quantitative PCR‐based assay that evaluates the faecal microbiota of dogs. This test quantifies the abundances of seven specific bacterial groups: *Faecalibacterium*, *Turicibacter*, *Blautia*, *Fusobacterium*, *Bifidobacterium*, *Bacteroides* and *Clostridium hiranonis*, and along with the total bacteria, these measurements are integrated into a single numerical value. Faecal SCFAs were measured by gas chromatography/mass spectrometry as described previously for dogs (Minamoto et al., 2019). Faecal long‐chain fatty acids, sterols and unconjugated BAs were measured by gas chromatography/mass spectrometry method as described for dogs (Galler et al., 2022). The gas chromatography/mass spectrometry analyses were performed at the gastrointestinal laboratory at Texas A&M.

### Health profile, serum fructosamine and blood haemoglobin A1c (HbA1c) measurements

The University of Illinois Veterinary Diagnostic Laboratory determined serum fructosamine concentrations and performed the analysis of a comprehensive health panel consisting of a complete blood count, serum biochemistry and urinalysis. Serum fructosamine was measured on Beckman Coulter DxC700 AU. The assay has inter‐assay CV% and intra‐assay CV% ranges of 2.78% to 4.45% and 4.04% to 8.04%, respectively, corresponding to a serum fructosamine range of 215 to 830 μmol/L. The fructosamine assay's linear range of detection is 10 to 1000 μmol/L. HbA1c concentrations from dried blood spots were measured by A1CARE (Baycom Diagnostics, Tallahassee, FL, USA) on a SPECTRAMAX PLUS immunoassay analyser at 720 nm. The assay has an average inter‐assay CV of 9.6% and an average intra‐assay CV of 3%. The assay's linear range of detection is 0.5% to 30%.

### Statistical analyses

Statistical analyses of clinical attributes and serum or faecal biomarkers were performed using SAS® OnDemand for Academics (SAS Institute Inc., Cary, NC, USA). The descriptive statistics of the dependent variables were described by the means and standard deviations. The data of the dependent variables were examined for normal distribution by inspection of Q‐Q plots, histogram and Shapiro–Wilk test. Variables that did not follow a normal distribution were log‐transformed. Analyses of variance of the dependent variables that had a normal or log‐normal distribution were performed with the MIXED procedure. The models included the fixed effects of treatment, period of the study, and the interaction between treatment and period. The model also included the dog as a random effect to account for repeated measures on the same dog over time. The marginal means and standard errors of the dependent variables for treatment, period and the combination between treatment and period were obtained and used for multiple mean comparisons using the Fisher's least significant difference test as implemented in the LSMEANS option of the MIXED procedure.

Regression lines of IG on week of measurement for each treatment were used with the MIXED procedure, including dog as a random effect. A *t*‐test was performed to determine if the slopes were statistically significantly different from each other.

Variables without normal or log‐normal distribution that instead had a Poisson distribution were analysed with the GLIMMIX procedure using a log link transformation with a model that included the same fixed and random effects as described above. Multiple mean comparisons were performed on the log scale. For all analyses, alpha error probability was set to P < 0.05.

## RESULTS

### IG concentration and total daily insulin dose

**FIG 4:**
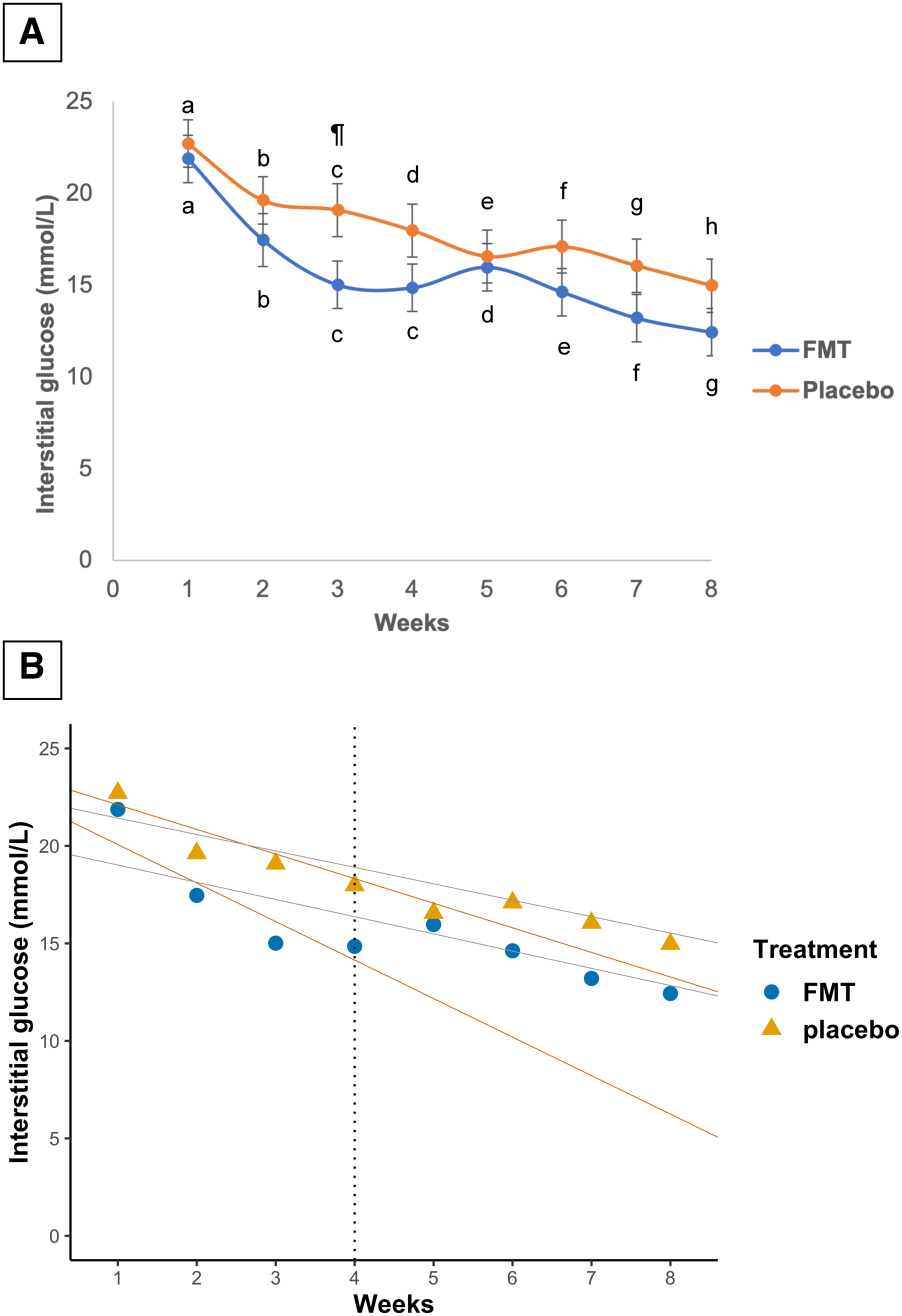
IG concentrations of dogs receiving lyophilised faeces or placebo. (A) Marginal means (±SE) IG concentrations of the FMT and placebo groups over the course of the study. (B) IG slopes of dogs receiving lyophilised faeces or placebo at the first 4 weeks of the study (orange) and during the 8 weeks of the study (grey). IG Interstitial glucose. Different superscript letters in (A) indicate significant differences in the marginal means of IG concentration within the group (P < 0.05). ¶ in (A) indicates a significant difference in the marginal mean of IG concentration between the groups (P = 0.035). The vertical dotted line in (B) marks the end of week 4.

**FIG 5:**
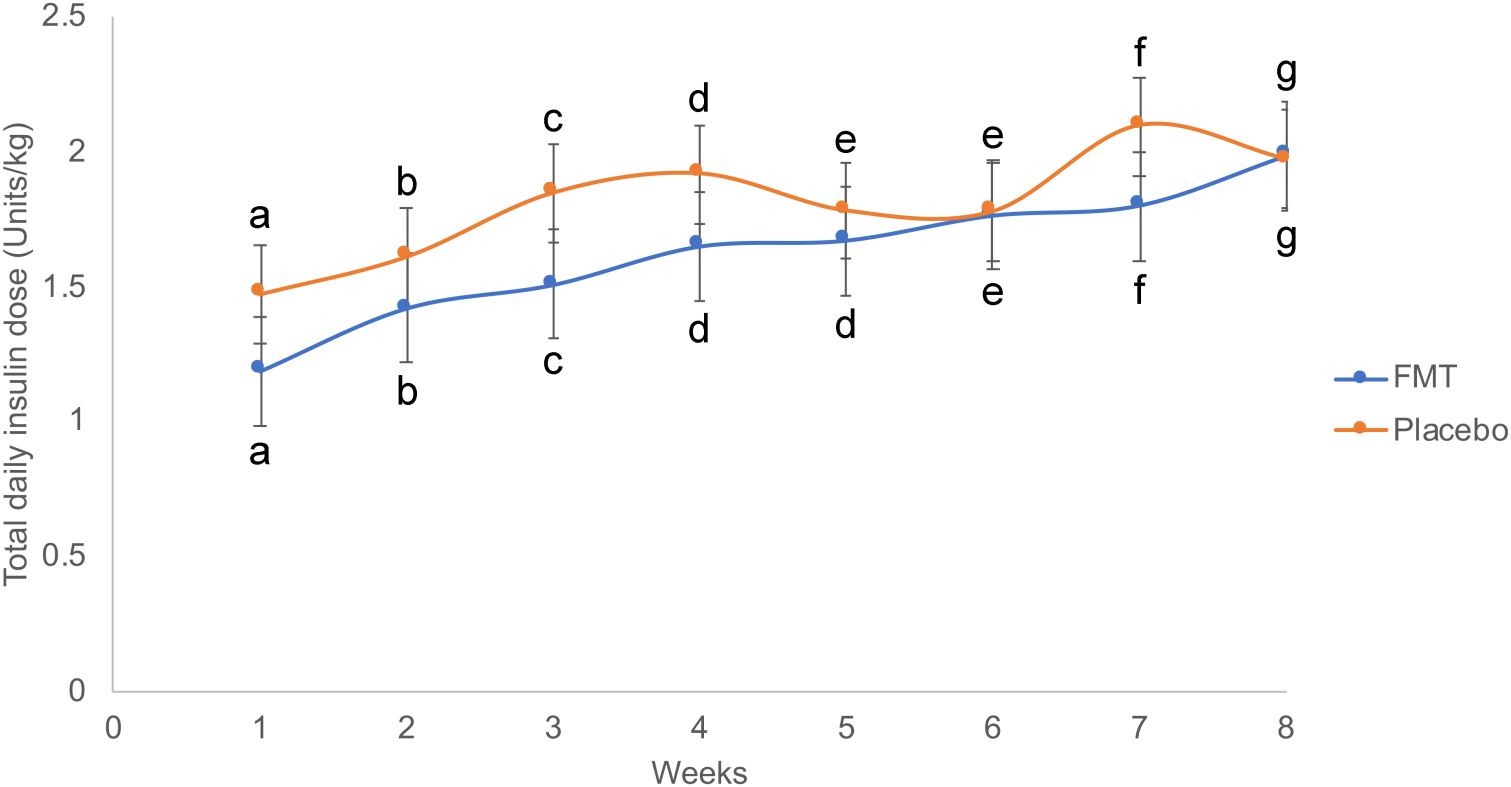
Marginal mean (±SE) total daily insulin dose (units/kg) of the FMT and placebo groups over the course of the study. Different letters indicate significant differences in the marginal means of daily insulin dose within group (P < 0.05).

The study's overall marginal mean (±SE) of the FMT group IG concentration (15.66 ± 1.28 mmol/L) did not differ from the placebo group (17.99 ± 1.44 mmol/L; P = 0.227). The within‐group IG concentration significantly decreased from week 1 to week 8 (P < 0.05; Fig 4A). The marginal mean (±SE) of the FMT group IG concentration at week 3 (14.99 ± 1.29 mmol/L) was significantly lower than the placebo group (19.07 ± 1.44 mmol/L; P = 0.035) but IG concentrations did not differ between groups at any other week. In the first 4 weeks of the study, the FMT group IG slope (±SE) (−1.97 ± 0.05) was significantly more negative than the placebo group (−1.26 ± 0.06; P < 0.001; Fig 4B) indicating a faster drop in FMT group's IG levels during that time. However, there were no differences between the groups' overall IG slopes (P = 0.163). The between‐dog and within‐dog CV% for IG were 16% and 84%, respectively. The study's overall marginal mean (±SE) of total daily insulin dose of the FMT group (1.619 ± 0.180 units/kg) did not differ significantly from the placebo group (1.806 ± 0.202 units/kg; P = 0.490; Fig 5). The within‐group marginal mean of total daily insulin dose significantly increased from week 1 to week 8 (P < 0.05) but it did not differ between groups at each of the study weeks. The between‐dog and within‐dog CV% for total daily insulin dose were 41% and 59%, respectively.

### 24‐hour water intake, serum HbA1c and serum fructosamine

Mean (±SD) 24‐hour water intake, serum HbA1c and serum fructosamine are present in Table 2 and Fig S1. The 24‐hour water intake was significantly lower in week 8 relative to baseline in the FMT group (P = 0.001) but not in the placebo group (P = 0.922) and was significantly lower in the FMT group relative to placebo in weeks 4 (P = 0.034), 6 (P = 0.046) and 8 (P = 0.002). There were no differences in the marginal means of serum HbA1c between the placebo and FMT groups at baseline (P = 0.366) or at the end of the study (P = 0.492). There were also no differences in the marginal means of serum fructosamine between placebo and FMT at any of the study timepoints, and serum fructosamine significantly decreased from baseline over the course of the study in both FMT and placebo (P = 0.01).

**Table 2:** Mean (±SD) serum HbA1c, serum fructosamine and 24‐hour water intake

Time	Treatment	HbA1c (%)	Fructosamine (μmol/L)	24 hour water intake (mL/kg/day)
Baseline	Placebo	5.25 ± 0.82	389.9 ± 87.22	104.33 ± 54.24
	FMT	4.84 ± 0.44	464.02 ± 95.2	74.5 ± 25.83
Week 2	Placebo		351.6 ± 96.33	119 ± 51.74
	FMT		427.32 ± 58.78	53.4 ± 14.47
Week 4	Placebo		331.53 ± 93.35	106.33 ± 55.19
	FMT		427.24 ± 80.19	42.4 ± 9.71
Week 6	Placebo		296.68 ± 59.63	86.67 ± 34.93
	FMT		387.66 ± 46.36	37.5 ± 17.45
Week 8	Placebo	3.98 ± 1.31	262.58 ± 104.24	113 ± 81.10
	FMT	3.42 ± 1	354.68 ± 54.11	25.68 ± 7.02

FMT Faecal microbiota transplantation, HbA1c Haemoglobin A1c

Population‐based reference interval for fructosamine: 172.0 to 292.0 μmol/L

Population‐based reference interval for HbA1c: 0% to 4%

### Gastrointestinal panel and serum C‐reactive protein

There were no significant differences between FMT and placebo in mean (±SD) serum TLI, cPLI, folate, cobalamin and CRP within and between groups over time (Table 3).

**Table 3:** Mean (±SD) serum CRP, cPLI, TLI, cobalamin and folate in FMT and placebo dogs at baseline and weeks 4 and 8

Time	Treatment	CRP (nmol/L)	cPLI (μg/L)	TLI (μg/L)	Cobalamin (pmol/L)	Folate (nmol/L)
Baseline	Placebo	101.86 ± 14.28	206.3 ± 178.9	28.5 ± 19	607.9 ± 100.3	36.6 ± 25.7
	FMT	94.25 ± 0.95	305.8 ± 385.1	48.2 ± 6.4	520.9 ± 229.4	22.5 ± 5.2
Week 4	Placebo	94.25 ± 0	132.8 ± 55.6	28.8 ± 18.4	678 ± 73	45.7 ± 21.6
	FMT	94.25 ± 0	221.6 ± 154.2	45.6 ± 7.5	531.2 ± 206.6	27 ± 7.3
Week 8	Placebo	106.62 ± 24.75	131.8 ± 37.3	24.8 ± 19.6	620.5 ± 112.1	40.7 ± 21.8
	FMT	94.25 ± 0	174.4 ± 131.3	42.7 ± 10.3	570.3 ± 240.5	27.7 ± 5.9

cPLI Canine pancreatic lipase immunoreactivity, CRP C‐reactive protein, FMT Faecal microbiota transplantation, TLI Trypsin‐like immunoreactivity

There were no significant differences between and within the FMT and placebo groups (P > 0.05)

Population‐based reference intervals for cPLI: 0 to 200 μg/L

Population‐based reference intervals for cTLI: 10.9 to 50.0 μg/L

Population‐based reference intervals for CRP: <10 mg/L

Population‐based reference intervals for cobalamin: 251 to 908 ng/L

Population‐based reference intervals for folate: 7.7 to 24.4 μg/L

### Serum GLP‐1 and faecal SCFA

The mean (±SD) GLP‐1 and faecal SCFA of the placebo and FMT diabetic dogs are presented in Tables 4 and 5. There were no significant differences in serum total GLP‐1 concentrations within or between the placebo and FMT groups at any time point. Dogs with DM had a significantly lower marginal mean (±SE) concentration of log‐transformed valerate (−0.78 ± 0.51 μmol/g) when compared to healthy dogs (1.5 ± 0.51 μmol/g; P = 0.006). The remainder of the SCFAs evaluated did not differ between healthy dogs and those with DM. The placebo group's marginal mean log‐transformed faecal butyrate concentration was significantly lower in week 8 than in weeks 0 and 4 (P = 0.03 and P = 0.002, respectively), and the FMT group's marginal mean log‐transformed faecal butyrate concentration was higher in week 8 than the placebo group's faecal butyrate concentration (P = 0.01). The placebo group's marginal mean log‐transformed faecal valerate concentration was significantly lower in week 8 than in week 4 (P = 0.03). There were no significant correlations between serum total GLP‐1 and faecal acetate (*r* = 0.42; P = 0.154), propionate (*r* = 0.25; P = 0.403) and butyrate (*r* = 0.21; P = 0.48).

**Table 4:** Mean (±SD) serum total GLP‐1 in FMT and placebo dogs at baseline and weeks 4 and 8

Time	Group	GLP‐1 (pM)
Baseline	Placebo	29.9 ± 11.2
	FMT	24.8 ± 11.8
Week 4	Placebo	33.3 ± 7.6
	FMT	24.3 ± 8.7
Week 8	Placebo	27.6 ± 11.6
	FMT	31.1 ± 9

FMT Faecal microbiota transplantation, GLP‐1 Glucagon‐like peptide 1

There were no significant differences between and within the FMT and placebo groups (P > 0.05)

**Table 5:** Mean (±SD) faecal SCFA (μmol/g) in FMT and placebo dogs at baseline and weeks 4 and 8

Metabolite (μmol/g)	Time	Group	Mean
Acetate	Baseline	Placebo	149 ± 16.4
		FMT	106.5 ± 37.7
	Week 4	Placebo	190.5 ± 81.5
		FMT	138.6 ± 54.1
	Week 8	Placebo	105.1 ± 17
		FMT	110.4 ± 39.1
Butyrate	Baseline	Placebo	18.3 ± 18
		FMT	24.6 ± 11.4
	Week 4	Placebo	75.4 ± 87.3
		FMT	14.5 ± 10.3
	Week 8^†^	Placebo	7.6 ± 11.7
		FMT	20.6 ± 8.9
Isobutyrate	Baseline	Placebo	3 ± 2.1
		FMT	3 ± 1.9
	Week 4	Placebo	5.2 ± 5.4
		FMT	3.7 ± 2.9
	Week 8	Placebo	5.9 ± 5.7
		FMT	3.6 ± 2.6
Isovalerate	Baseline	Placebo	3.2 ± 1.5
		FMT	5.8 ± 3.8
	Week 4	Placebo	7 ± 7
		FMT	7.2 ± 5.4
	Week 8	Placebo	11.8 ± 10.7
		FMT	6.6 ± 5.6
Propionate	Baseline	Placebo	97.7 ± 55.1
		FMT	32.6 ± 22
	Week 4	Placebo	105.1 ± 101
		FMT	82.3 ± 92
	Week 8	Placebo	59.8 ± 42.2
		FMT	52.1 ± 61.1
Valerate	Baseline	Placebo	1.9 ± 2.8
		FMT	0.6 ± 0.6
	Week 4	Placebo	6.2 ± 3
		FMT	2 ± 3.6
	Week 8	Placebo	0.3 ± 0.2
		FMT	3.9 ± 7.9

FMT Faecal microbiota transplantation; SCFA Short chain fatty acids

^†^ Placebo's faecal butyrate in week 8 was significantly lower than FMT in week 8 and placebo in baseline and week 4 (P < 0.05)

### Faecal BAs, fatty acids and sterols

The mean (±SD) faecal BAs, faecal fatty acids and faecal sterols of the healthy control dogs and the diabetic dogs are presented in Tables 6 and 7. The diabetic dogs had significantly lower marginal mean concentrations of fusosterol (P = 0.045), log‐transformed betasitosterol (P = 0.030), log‐transformed sitostanol (P = 0.004) and log‐transformed stigmasterol (P = 0.037) and a higher concentration of butylarachidonate (P = 0.001).

**Table 6:** Mean (±SD) faecal fatty acids and sterols of diabetic dogs and healthy controls

Metabolite (μg/mg)	Group	Mean	SD
Total fatty acids	DM	30.23	21.36
	Control	21.31	5.93
Total sterols	DM	6.44	4.72
	Control	6.54	2.43
TMS versus chol cholesterol	DM	3.81	3.79
	Control	2.34	1.07
TMS betasitosterol	DM	1.28	0.83
	Control	2.1	0.65
TMS brassicasterol	DM	0.02	0.03
	Control	0.01	0.01
TMS campesterol	DM	0.44	0.35
	Control	0.57	0.17
TMS cholestanol	DM	0.17	0.15
	Control	0.11	0.05
TMS coprostanol	DM	0.17	0.36
	Control	0.02	0.01
TMS fusosterol	DM	0.05	0.03
	Control	0.08	0.03
TMS lathosterol	DM	0.03	0.03
	Control	0.02	0.02
TMS sitostanol	DM	0.3	0.29
	Control	1.02	0.64
TMS stigmasterol	DM	0.18	0.12
	Control	0.29	0.1
Butylalphalinolenate	DM	1.29	1.7
	Control	0.52	0.59
Butylarachidonate	DM	4.77	7.89
	Control	1.32	0.32
Butylcisvaccenate	DM	0.8	0.92
	Control	0.92	0.52
Butyldocosanoate	DM	0.14	0.06
	Control	0.12	0.03
Butylerucate	DM	0.02	0.01
	Control	0.01	0.01
Butylgondoate	DM	0.17	0.09
	Control	0.03	0.03
Butyllinoleate	DM	6.69	5.34
	Control	4.75	1.6
Butylmyristate	DM	0.23	0.14
	Control	0.35	0.18
Butylnervonate	DM	0.09	0.06
	Control	0.05	0.03
Butyloleate	DM	4.99	2.61
	Control	4.3	0.82
Butylpalmitate	DM	6.41	4.14
	Control	5.54	2.01
Butylstearate	DM	4.64	4.74
	Control	3.41	2.37

DM Diabetes mellitus

**Table 7:** Mean (±SD) faecal BA of diabetic dogs and healthy controls

Metabolite (ng/mg)	Group	Mean	SD
TBA	DM	8525.44	7203.71
	Control	6032	3858.97
TPBA	DM	2360.67	4401.23
	Control	747.22	608.75
% PBA	DM	29.33	32.55
	Control	11.67	7.3
TSBA	DM	6164.78	4175.6
	Control	5284.78	3442.49
% SBA	DM	70.67	32.55
	Control	88.33	7.3
Chenodeoxycholic acid	DM	1582	4408.65
	Control	121.11	87.29
% Chenodeoxycholic acid	DM	8.04	16.77
	Control	1.79	1.17
Cholic acid	DM	778.89	688.6
	Control	626.33	537.33
% Cholic acid	DM	21.3	29.95
	Control	9.92	6.4
Deoxycholic acid	DM	5170.22	3433.38
	Control	4358.67	3185.89
% Deoxycholic acid	DM	60.72	30.27
	Control	67.78	11.38
Lithocholic acid	DM	921.56	1075.38
	Control	795.78	338.75
% Lithocholic acid	DM	8.69	5.46
	Control	17.78	10.21
Ursodeoxycholic acid	DM	72.89	30.69
	Control	130.22	98.53
% Ursodeoxycholic acid	DM	1.27	0.82
	Control	2.73	2.32

TBA Total bile acid, TPBA Total primary bile acid, TSBA Total secondary bile acid

The diabetic dogs had significantly higher marginal mean log‐transformed total primary BAs (P < 0.001), log‐transformed chenodeoxycholic acid (P < 0.001), log‐transformed cholic acid (P < 0.001), % log‐transformed primary BAs (P < 0.001), % log‐transformed chenodeoxycholic acid (P < 0.001) and % log‐transformed cholic acid (P < 0.001) than the healthy control group. The diabetic dogs also had significantly higher marginal mean lithocholic acid (P < 0.001) and lower % lithocholic acid (P = 0.032), % SBAs (P = 0.001) and log‐transformed % ursodeoxycholic acid (0.047) than the healthy control group. The mean (±SD) faecal fatty acids, faecal sterols and faecal BAs of the FMT and placebo dogs are presented in Tables 8 and 9. The FMT group had significantly lower marginal mean concentrations of log‐transformed faecal sitostanol than placebo at baseline (P = 0.022), 4 weeks (P = 0.010) and 8 weeks (P = 0.013). The FMT group had significantly lower marginal mean concentrations of log‐transformed faecal total primary BAs at week 8 compared with the placebo group (P = 0.035).

**Table 8:** Mean (±SD) faecal fatty acids and sterols of FMT and placebo dogs at baseline, 4 and 8 weeks

Metabolite (μg/mg)	Timepoint	Group	Mean	SD
Total fatty acids	1	FMT	22.04	6.09
		Placebo	40.48	30.26
	2	FMT	19.08	8.64
		Placebo	20.53	12.01
	3	FMT	24.94	10.03
		Placebo	21.32	8.69
Total sterols	1	FMT	5.62	2.46
		Placebo	7.48	6.99
	2	FMT	3.92	1.56
		Placebo	7.43	5.84
	3	FMT	6.24	2.72
		Placebo	8.23	3.37
TMS versus chol cholesterol	1	FMT	3.46	2.7
		Placebo	4.24	5.31
	2	FMT	1.87	1.27
		Placebo	3.56	4.28
	3	FMT	3.79	2.79
		Placebo	4.54	3.85
TMS betasitosterol	1	FMT	0.97	0.54
		Placebo	1.66	1.06
	2	FMT	1.07	0.29
		Placebo	1.9	0.82
	3	FMT	1.19	0.85
		Placebo	1.75	0.58
TMS brassicasterol	1	FMT	0.01	0
		Placebo	0.04	0.05
	2	FMT	0.01	0
		Placebo	0.02	0.01
	3	FMT	0.02	0.01
		Placebo	0.02	0.01
TMS campesterol	1	FMT	0.31	0.07
		Placebo	0.6	0.51
	2	FMT	0.3	0.08
		Placebo	0.63	0.31
	3	FMT	0.4	0.21
		Placebo	0.58	0.11
TMS cholestanol	1	FMT	0.17	0.13
		Placebo	0.16	0.19
	2	FMT	0.11	0.05
		Placebo	0.18	0.14
	3	FMT	0.26	0.19
		Placebo	0.22	0.13
TMS coprostanol	1	FMT	0.3	0.46
		Placebo	0.02	0.02
	2	FMT	0.14	0.26
		Placebo	0.2	0.38
	3	FMT	0.16	0.2
		Placebo	0.19	0.37
TMS fusosterol	1	FMT	0.04	0.02
		Placebo	0.06	0.03
	2	FMT	0.04	0.01
		Placebo	0.06	0.03
	3	FMT	0.05	0.03
		Placebo	0.07	0.02
TMS lathosterol	1	FMT	0.03	0.02
		Placebo	0.04	0.04
	2	FMT	0.01	0.01
		Placebo	0.02	0.02
	3	FMT	0.02	0.01
		Placebo	0.03	0.02
TMS sitostanol	1	FMT	0.18	0.23
		Placebo	0.46	0.31
	2	FMT	0.18	0.24
		Placebo	0.55	0.28
	3	FMT	0.16	0.11
		Placebo	0.63	0.37
TMS stigmasterol	1	FMT	0.15	0.08
		Placebo	0.22	0.15
	2	FMT	0.16	0.05
		Placebo	0.34	0.22
	3	FMT	0.21	0.18
		Placebo	0.2	0.07
Butylalphalinolenate	1	FMT	1.17	1.79
		Placebo	1.44	1.85
	2	FMT	2.77	2.05
		Placebo	0.27	0.17
	3	FMT	1.3	1.7
		Placebo	0.39	0.59
Butylarachidonate	1	FMT	2.86	3.49
		Placebo	7.16	11.66
	2	FMT	1.1	0.42
		Placebo	1.69	1.53
	3	FMT	2.72	2.61
		Placebo	1.77	0.9
Butylcisvaccenate	1	FMT	1.14	1.13
		Placebo	0.38	0.37
	2	FMT	0.51	0.35
		Placebo	1.08	1.62
	3	FMT	1.51	1.4
		Placebo	0.87	0.25
Butyldocosanoate	1	FMT	0.12	0.06
		Placebo	0.16	0.05
	2	FMT	0.09	0.02
		Placebo	0.2	0.17
	3	FMT	0.15	0.08
		Placebo	0.19	0.16
Butylerucate	1	FMT	0.02	0.01
		Placebo	0.02	0.01
	2	FMT	0.02	0.01
		Placebo	0.03	0.02
	3	FMT	0.01	0.01
		Placebo	0.03	0.01
Butylgondoate	1	FMT	0.15	0.1
		Placebo	0.19	0.09
	2	FMT	0.11	0.09
		Placebo	0.1	0.03
	3	FMT	0.1	0.09
		Placebo	0.06	0.06
Butyllinoleate	1	FMT	4.3	2.42
		Placebo	9.67	6.85
	2	FMT	4.61	3.93
		Placebo	3.78	1.61
	3	FMT	4.41	1.7
		Placebo	3.09	1.79
Butylmyristate	1	FMT	0.22	0.15
		Placebo	0.25	0.16
	2	FMT	0.18	0.12
		Placebo	0.22	0.1
	3	FMT	0.22	0.2
		Placebo	0.35	0.09
Butylnervonate	1	FMT	0.1	0.07
		Placebo	0.08	0.05
	2	FMT	0.06	0.03
		Placebo	0.06	0.04
	3	FMT	0.07	0.06
		Placebo	0.06	0.03
Butyloleate	1	FMT	4.15	1.32
		Placebo	6.03	3.64
	2	FMT	3.47	2.35
		Placebo	3.29	0.93
	3	FMT	4.21	0.81
		Placebo	3.13	1.06
Butylpalmitate	1	FMT	4.86	1.64
		Placebo	8.35	5.76
	2	FMT	4.11	1.06
		Placebo	4.79	2.43
	3	FMT	5.55	1.78
		Placebo	6.19	2.63
Butylstearate	1	FMT	2.94	1.87
		Placebo	6.76	6.67
	2	FMT	2.06	0.85
		Placebo	5.04	6.91
	3	FMT	4.66	4.46
		Placebo	5.19	4.09

**Table 9:** Mean (±SD) faecal bile acids of FMT and placebo dogs at baseline, 4 and 8 weeks

Metabolite (ng/mg)	Timepoint	Group	Mean	SD
TBA	Baseline	FMT	10,761.8	8852.36
		Placebo	5730	3891.27
	4 weeks	FMT	6278.6	4076.55
		Placebo	9390.5	3405.97
	8 weeks	FMT	7365.8	4955.44
		Placebo	13,549.75	5310.05
TPBA	Baseline	FMT	3502.6	5896.1
		Placebo	933.25	643.1
	4 weeks	FMT	1170.4	2269.91
		Placebo	665	478.13
	8 weeks	FMT	949.2	1325.48
		Placebo	7437.5	7806.4
% PBA	Baseline	FMT	30.8	38.23
		Placebo	27.5	29.46
	4 weeks	FMT	22	39.94
		Placebo	9.75	10.84
	8 weeks	FMT	23	25.14
		Placebo	51.75	39
TSBA	Baseline	FMT	7259.2	4518.65
		Placebo	4796.75	3844.37
	4 weeks	FMT	5108.4	4855.64
		Placebo	8725.75	3706.52
	8 weeks	FMT	6416.2	4956.57
		Placebo	6112.25	5490.37
% SBA	Baseline	FMT	69.2	38.23
		Placebo	72.5	29.46
	4 weeks	FMT	78	39.94
		Placebo	90.25	10.84
	8 weeks	FMT	77	25.14
		Placebo	48.25	39
Chenodeoxycholic acid	Baseline	FMT	2742.4	5921.94
		Placebo	131.5	145.7
	4 weeks	FMT	97.4	155.77
		Placebo	92.75	55.04
	8 weeks	FMT	195	257.71
		Placebo	1621.25	2148.87
% Chenodeoxycholic acid	Baseline	FMT	12.3	22.5
		Placebo	2.73	2.6
	4 weeks	FMT	1.74	2.74
		Placebo	1.28	1.18
	8 weeks	FMT	4.88	6.34
		Placebo	11.3	13.25
Cholic acid	Baseline	FMT	760.6	864.09
		Placebo	801.75	517.36
	4 weeks	FMT	1072.8	2116.36
		Placebo	572.25	425.89
	8 weeks	FMT	754.2	1251.85
		Placebo	5816.25	8253.27
% Cholic acid	Baseline	FMT	18.64	34.32
		Placebo	24.63	28.19
	4 weeks	FMT	20.26	37.44
		Placebo	8.55	9.62
	8 weeks	FMT	17.96	23.96
		Placebo	40.43	43.62
Deoxycholic acid	Baseline	FMT	6090.4	3642.17
		Placebo	4020	3251.52
	4 weeks	FMT	4124.6	3596.28
		Placebo	7636	3195.02
	8 weeks	FMT	4937	3471.15
		Placebo	4820.75	4337.87
% Deoxycholic acid	Baseline	FMT	60.58	37.03
		Placebo	60.9	24.78
	4 weeks	FMT	65.68	34.25
		Placebo	79.15	8.12
	8 weeks	FMT	60.46	20.42
		Placebo	38.68	30.67
Lithocholic acid	Baseline	FMT	1086.4	1404.97
		Placebo	715.5	591.64
	4 weeks	FMT	912.4	1295.07
		Placebo	957.75	637.66
	8 weeks	FMT	1383.2	1921.8
		Placebo	1131.75	1228.82
% Lithocholic acid	Baseline	FMT	7.52	5.37
		Placebo	10.15	5.99
	4 weeks	FMT	10.8	9.01
		Placebo	9.63	4.79
	8 weeks	FMT	14.5	13.77
		Placebo	8.5	9.03
Ursodeoxycholic acid	Baseline	FMT	82	38.01
		Placebo	61.5	16.54
	4 weeks	FMT	71.4	33.47
		Placebo	132	99.04
	8 weeks	FMT	96.2	92.58
		Placebo	159.75	118.44
% Ursodeoxycholic acid	Baseline	FMT	1	0.5
		Placebo	1.6	1.09
	4 weeks	FMT	1.52	1.46
		Placebo	1.38	0.74
	8 weeks	FMT	2.18	2.64
		Placebo	1.1	0.43

TBA Total bile acid, TPBA Total primary bile acid, TSBA Total secondary bile acid

### Faecal DI

**FIG 6:**
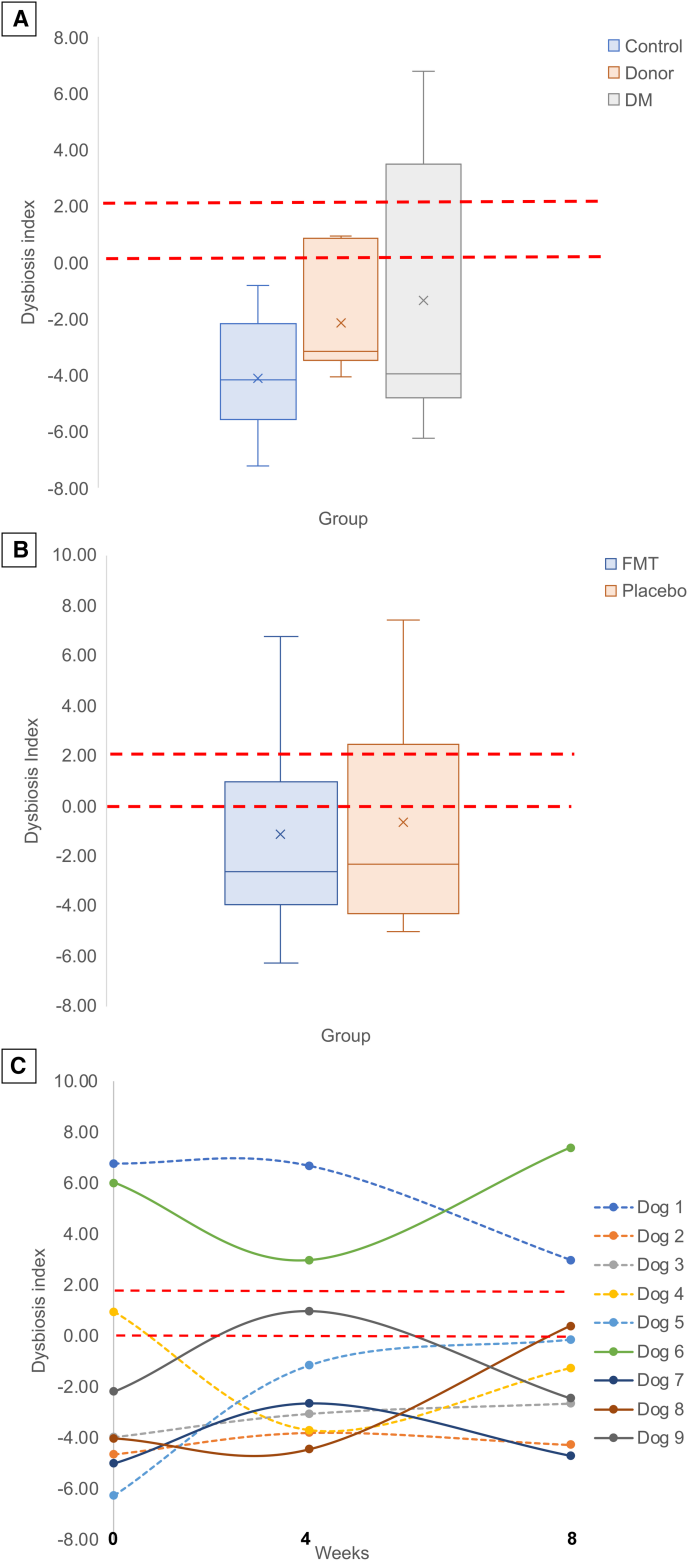
Dysbiosis index (DI). (A) Boxplots of DI of dogs with DM, healthy controls and FMT donors. (B) Boxplots of DI of dogs in the FMT and placebo groups. (C) DI of individual dogs from the FMT and placebo groups at admission, 4 and 8 weeks. Dotted lines indicate FMT. The red dashed lines represent the range of the high end of the DI reference interval. The box represents the 25th and 75th percentiles (IQR), the horizontal line represents the median, and the × represents the mean. The whiskers represent the maximum and minimum values below and above the upper (75th percentile + IQR) and lower (and 25th percentile − IQR) fences, respectively.

There were no significant differences between the faecal DI marginal means (±SE) of the diabetic dogs (−1.36 ± 1.13), the healthy controls (−4.11 ± 1.13) and the donor dogs (−2.16 ± 1.28; P = 0.233; Fig 6A) and the marginal means were all within the reported Gastrointestinal Laboratory reference interval (<0). There were no significant differences between the faecal DI marginal means (±SE) of the FMT dogs (−1.15 ± 1.80), the placebo dogs (−0.63 ± 2.01; Fig 6B) and they were all within the reported Gastrointestinal Laboratory reference interval (<0). The between‐dog and within‐dog CV% for DI were 73% and 27%, respectively (Fig 6C).

There were significant differences in the faecal DI marginal mean (±SE) of log DNA *Faecalibacterium* of the diabetic dogs (4.9 ± 0.44 ng/μL; P = 0.002) compared to the donor dogs (7.21 ± 0.5 ng/μL), and between the donor dogs and healthy control dogs (5.7 ± 0.44 ng/μL; P = 0.033), but there were no significant differences between the healthy control dogs and diabetic dogs (P = 0.215). The faecal DI marginal mean (±SE) of log DNA *Faecalibacterium* of the FMT dogs was significantly higher than that of the placebo dogs in week 4 (6.87 ± 0.45 ng/μL vs. 5.41 ± 0.5 ng/μL; P = 0.046) but not at week 8 (6.11 ± 0.45 ng/μL vs. 4.86 ± 0.5 ng/μL; P = 0.083).

The reported Gastrointestinal Laboratory reference interval for log DNA *Faecalibacterium* is 3.4 to 8 ng/μL.

The faecal DI marginal mean (±SE) of log DNA *Streptococcus* of the diabetic dogs (5.06 ± 0.58 ng/μL) did not differ from the donor dogs (5.38 ± 0.66 ng/μL; P = 0.715) but both significantly differed from the healthy control dogs (2.98 ± 0.58 ng/μL; P = 0.019 and P = 0.012, respectively). The reported Gastrointestinal Laboratory reference interval for log DNA *Streptococcus* is 1.9 to 8 ng/μL.

The faecal DI marginal means (±SE) of log DNA of *Fusobacterium* and *Escherichia coli* of the donor dogs (9.66 ± 0.30 ng/μL and 6.52 ± 0.56 ng/μL) were significantly higher than healthy control dogs (8.43 ± 0.26 ng/μL and 4.90 ± 0.49 ng/μL; P = 0.005 and P = 0.042), and both did not significantly differ from the diabetic dogs (9.09 ± 0.26 ng/μL and 6.11 ± 0.49 ng/μL; P > 0.05). The reported Gastrointestinal Laboratory reference interval for log DNA *Fusobacterium* and *E. coli* is 7.0 to 10.3 ng/μL and 0.9 to 8.0 ng/μL.

## DISCUSSION

In this study, we hypothesised that FMT would affect the gut microbiome of diabetic dogs in a manner that will improve glycaemic control by increasing the abundance of SCFA producing bacteria and faecal SCFA concentrations, decreasing the abundance of proinflammatory diabetogenic bacteria, altering bacterial BA metabolism and enhancing incretin hormone activity. In this randomised, placebo‐controlled, double‐blinded prospective study, administering lyophilised faecal capsules to diabetic dogs for 2 months had a minimal, clinically insignificant effect on glycaemic control in the FMT diabetic dog group. Two FMT‐associated effects we observed included a more negative 4‐week IG slope (i.e. a faster decline in IG levels) and a lower 24‐hour water intake compared to the placebo group. These FMT‐associated effects were noted despite a small number of study participants and a very small effect size (see below).

We found that there were no significant differences in the mean DI between diabetic dogs, healthy controls and donor dogs, nor between the FMT and placebo dog groups throughout the duration of the study. However, there was a large between‐dog CV% in the DI, because while nine dogs had a normal DI, two dogs (one in the FMT and one in the placebo group) had a DI that was outside of the population‐based reference interval for the duration of the study. Therefore, our findings indicate that in this cohort of dogs, DM was not associated with a profound gut dysbiosis; however, significant differences in abundances of certain bacterial genera (e.g. SCFA producers; see below) might be amenable to correction with FMT and thus may have a beneficial therapeutic effect on DM. Nevertheless, we surmise that a small percentage of dogs with DM might have a significant increase in the DI, which may indicate an underlying chronic enteropathy in addition to the DM. It is unknown whether these dogs would have a different response to FMT compared with dogs DI within the population‐based reference interval. Because increased DI was not present in all dogs at the time of enrolment, it is also possible that the effect of FMT could have been stronger had we only included diabetic dogs with a pre‐FMT DI outside of the population‐based reference interval. Additional studies are needed to determine how measuring DI impacts the prognosis for normalisation of microbiota, responses to FMT, and whether additional treatment for an underlying chronic enteropathy may be needed.

The SCFA fermenter *Faecalibacterium prausnitzii* has been previously shown to be important for glucose tolerance and has been decreased in abundance in diabetic humans (Wang et al., 2020). *Faecalibacterium prausnitzii* is an important butyrate producer that has been positively linked to improved gut mucosal barrier and glucose tolerance (Ferreira‐Halder et al., 2017; Tilg & Moschen, 2014). Our study demonstrated that following FMT, the DI's marginal mean log DNA of *Faecalibacterium* increased in week 4 in the FMT group compared to the placebo group and was higher (though not significantly different) in week 8. Furthermore, the FMT group had a higher faecal butyrate concentration at week 8 compared to placebo. These findings suggest that, like rodents and humans (Chen et al., 2023; de Groot et al., 2021; Han et al., 2021; Wang et al., 2019; Wu et al., 2022), FMT in dogs may have some potential to increase the abundance of bacteria that are considered beneficial to glucose tolerance and increase faecal SCFA content and therefore potentially offer some benefits for glucose regulation through SCFA signalling (Kang et al., 2024).

In this study, we measured total GLP‐1 concentrations in the serum. Most of the GLP‐1 in the blood is a biologically inactive form of GLP‐1. The active incretin hormone acts in a paracrine and endocrine manner and has a short half‐life of approximately 2 minutes, as it is rapidly inactivated by dipeptidyl peptidase‐4 and neutral endopeptidase 24.11. The measurement of total GLP‐1 represents this incretin hormone's secretory capacity, and in our study, it was not affected by FMT as there were no differences in total GLP‐1 concentrations between groups. For a similar reason, we did not find significant correlations between total GLP‐1 and SCFAs despite the latter being known as a potent GLP‐1 secretagogues. Future studies focusing on the pharmacodynamic effect of incretin hormones or using methodology that will capture the biologically active GLP‐1 will be required to ascertain the effect that FMT might have on canine incretin hormones in diabetes.

We also found that diabetic dogs had lower faecal concentrations of several phytosterols, including betasitosterol, sitostanol and stigmasterol, and that FMT resulted in a lower concentration of sitostanol. Dietary phytosterols can reduce blood cholesterol levels and have the potential to be useful in diabetes through decreasing insulin resistance (Salehi‐Sahlabadi et al., 2020). The reason for the phytosterol differences between groups is unclear, and we speculate that it could be due to a difference in dietary phytosterol content between regular dog diets and diabetic dog diets, differences in intestinal transit time between diabetic and nondiabetic dogs, or due to increased phytosterol metabolism in diabetic dogs. Further studies that will determine the concentrations of phytosterols in the diet, blood and faeces will be required to ascertain what role, if at all, phytosterols play in canine diabetes.

Consistent with a previous diabetic dog study (Jergens et al., 2019), we observed alterations in the faecal BA profile of diabetic subjects compared to healthy controls. Diabetic dogs exhibited increased total and percentage primary BAs, as well as changes in individual BAs (higher absolute and percentage cholic and chenodeoxycholic acids and higher absolute lithocholic acid), accompanied by a decrease in percentage SBAs, lithocholic acid and ursodeoxycholic acid. Although we did not measure circulating BAs, these faecal changes raise the possibility that the systemic BA pool may have been similarly affected. Importantly, our analysis also revealed that FMT had a modest but notable effect on the BA pool, reducing total primary BAs at week 8 compared to the placebo group. The degree to which shifts in the BA profile mediate the global effects of FMT remains uncertain, but these findings support the notion that modulating the gut microbiome could influence BA metabolism and, potentially, host metabolic regulation.

The study has several limitations. The first limitation is the small number of study participants. Despite our best efforts to enrol patients, which included advertisement of the study in social media and interviews in local radio and TV networks, we managed to enrol only nine dogs. However, this was not enough to demonstrate clinically significant lasting differences in glycaemic control between groups. To estimate the sample size required to demonstrate differences in IG levels between FMT and placebo groups in a study with a similar design as ours, we first calculated the effect size (*r* = 0.006) with an online effect size calculator (https://lbecker.uccs.edu/) which required the *t*‐value (−1.39) and the degrees of freedom (43 × 10^3^) retrieved from the analysis of the IG slopes of the 8‐week period of our study. Then, using G*Power 3.1.9.6 software for power‐sample size estimation (Faul et al., 2007, 2009), we estimated the sample size for our study design (i.e. linear mixed effect model with repeated measures consisting of one response variable, three predictors and two groups), with a power of 0.8 and an alpha error probability of 0.05. We found that a total number of 1822 diabetic dogs would be required to detect a significant difference in IG levels between the FMT and placebo groups. Hence, in the future, if FMT should be investigated in a prospective randomised blinded controlled study setting for diabetes, it will have to be a multi‐centre effort to overcome recruitment challenges and gain enough power to detect a small effect size similar to the one in our study. Secondly, in the human medicine field of FMT, both single donors and pools of donors have been shown to lead to successful and failed FMT, and the predictors of a successful FMT are currently not well characterised and are dependent on both donor and recipient factors. A similar information does not exist in the veterinary literature. We did not have a 1:1 donor‐to‐recipient ratio as we initially intended because of an unexpected shortage in lyophilised faecal material. We acknowledge that it resulted in our inability to assess individual donors, but we also argue that even if we had individual donors for the recipient, it would have still been difficult to assess their individual effect because of the small number of recipient dogs. Lastly, in the context of a controlled clinical study, where numerous interacting variables can yield opposing influences, the notable age differences between healthy and diabetic dogs may have contributed to an unforeseen degree of confounding. Since both age and breed have been linked to alterations in the gut microbiota (You & Kim, 2021), these elements warrant acknowledgment as potential confounders in the interpretation of our findings.

With regard to the differences in 24‐hour water intake between the FMT and placebo groups, we acknowledge that the small sample sizes and missing data points make the analysis susceptible to Type I error (i.e. incorrectly rejecting the null hypothesis). We also recognise that external factors, such as changes in the dogs' environment, undiagnosed comorbidities or owner‐introduced measurement errors, could have influenced the water intake data. Notably, one dog in the placebo group was an influential outlier (Fig S1) with a high water intake (>100 mL/kg/day), which increased the mean 24‐hour water intake for that group. Based on the owners' report of excellent glycaemic control at each visit, we suspect measurement errors may have occurred. Over the 2‐month study, this dog required a mean (±SD) total daily insulin dose of 1.73 ± 0.75 units/kg (Toujeo), had a mean (±SD) IG of 344 ± 142 with a CV% of 41% and showed a drop in HbA1c from 5.5% to 3.3%. Even when excluding this outlier, a significant difference in water intake remained between groups (P = 0.005). We want to end the discussion on the 24‐hour water intake by proposing to the readers the possibility that the observed differences between groups may represent a genuine biological effect through a non‐osmotic diuretic mechanism. For instance, if FMT increases the fraction of biologically active GLP‐1, it may suppress thirst mechanisms in the brain and thereby reduce water intake, as seen in healthy humans subjected to injections of GLP‐1 analogues (Winzeler et al., 2020).

This study provides proof of concept for using FMT in canine diabetes. It remains to be determined whether a clinically relevant FMT‐associated impact might emerge over a longer treatment duration when FMT is combined in a multimodal strategy with insulin and a DM‐specific diet. The small effect size of FMT on IG in this study should be considered when designing future FMT studies for canine DM. Further investigation is required to determine the various biological mechanisms by which FMT could impact canine DM.

### Author contributions

**R. Brown:** Conceptualization (equal); data curation (lead); formal analysis (equal); investigation (equal); methodology (equal); project administration (lead); writing – original draft (lead); writing – review and editing (equal). **P. Barko:** Conceptualization (equal); data curation (equal); formal analysis (equal); methodology (equal); software (equal); writing – review and editing (equal). **J. D. J. Ruiz Romero:** Data curation (equal); investigation (equal); project administration (equal); writing – review and editing (equal). **D. A. Williams:** Conceptualization (equal); methodology (equal); writing – review and editing (equal). **A. Gochenauer:** Conceptualization (equal); project administration (equal); writing – review and editing (equal). **J. Nguyen:** Investigation (equal); writing – review and editing (equal). **J. S. Suchodolski:** Conceptualization (equal); data curation (equal); methodology (equal); resources (equal); writing – review and editing (equal). **R. Pilla:** Data curation (equal); writing – review and editing (equal). **H. Ganz:** Methodology (equal); resources (equal); writing – review and editing (supporting). **N. Lopez‐Villalobos:** Formal analysis (equal); software (equal); writing – review and editing (equal). **A. Gal:** Conceptualization (lead); data curation (lead); formal analysis (equal); funding acquisition (lead); investigation (lead); methodology (lead); project administration (lead); resources (lead); supervision (lead); writing – original draft (lead); writing – review and editing (lead).

### Funding information

This research received partial funding from the University of Illinois at Urbana‐Champaign Companion Animal Research Grant Program's Vera Arvilla Traxler Fund.

### Conflict of interest

All authors other than Dr. Holly Ganz declare no conflict of interest. Dr. Ganz works for AnimalBiome, which is the company that donated the lyophilised donor faeces. Dr. Ganz was not involved in the data analyses and did not influence in any way the study design, its execution nor the conclusions of this research.

## Supporting information


**Fig S1.** 24‐hour water intake jitter boxplots. The box represents the 25th and 75th percentiles (IQR), the horizontal line represents the median, and the × represents the mean. The whiskers represent the maximum and minimum values below and above the upper (75th percentile + IQR) and lower (and 25th percentile − IQR) fences, respectively. Individual black dots represent individual dogs

**Table S1.** Dietary nutritional analysis of healthy and diabetic dog study participants

## Data Availability

Data presented in this study are available on request from the corresponding author.

## References

[jsap13865-bib-0001] Baioni, E. , Scanziani, E. , Vincenti, M.C. , Leschiera, M. , Bozzetta, E. , Pezzolato, M. et al. (2017) Estimating canine cancer incidence: findings from a population‐based tumour registry in northwestern Italy. BMC Veterinary Research, 13, 203. 28659149 10.1186/s12917-017-1126-0 PMC5490209

[jsap13865-bib-0002] Chang, C.H. , Lidbury, J.A. , Suchodolski, J.S. & Steiner, J.M. (2022) Effect of oral or injectable supplementation with cobalamin in dogs with hypocobalaminemia caused by chronic enteropathy or exocrine pancreatic insufficiency. Journal of Veterinary Internal Medicine, 36, 1607–1621. 36054643 10.1111/jvim.16528 PMC9511088

[jsap13865-bib-0003] Chen, L. , Guo, L. , Feng, S. , Wang, C. , Cui, Z. , Wang, S. et al. (2023) Fecal microbiota transplantation ameliorates type 2 diabetes via metabolic remodeling of the gut microbiota in db/db mice. BMJ Open Diabetes Research & Care, 11, e003282. Available from: 10.1136/bmjdrc-2022-003282 PMC10230930 37253485

[jsap13865-bib-0004] Corradini, S. , Pilosio, B. , Dondi, F. , Linari, G. , Testa, S. , Brugnoli, F. et al. (2016) Accuracy of a flash glucose monitoring system in diabetic dogs. Journal of Veterinary Internal Medicine, 30, 983–988. 27318663 10.1111/jvim.14355 PMC5094557

[jsap13865-bib-0005] Covin, M.A. , Gomez, R.R. , Suchodolski, J.S. , Steiner, J.M. & Lidbury, J.A. (2021) Analytical validation of a point‐of‐care test and an automated immunoturbidimetric assay for the measurement of canine C‐reactive protein in serum. Canadian Journal of Veterinary Research, 85, 285–292. 34602733 PMC8451710

[jsap13865-bib-0006] Davison, L.J. , Herrtage, M.E. & Catchpole, B. (2005) Study of 253 dogs in the United Kingdom with diabetes mellitus. Veterinary Record, 156, 467–471. 15828742 10.1136/vr.156.15.467

[jsap13865-bib-0007] de Groot, P. , Nikolic, T. , Pellegrini, S. , Sordi, V. , Imangaliyev, S. , Rampanelli, E. et al. (2021) Faecal microbiota transplantation halts progression of human new‐onset type 1 diabetes in a randomised controlled trial. Gut, 70, 92–105. 33106354 10.1136/gutjnl-2020-322630 PMC7788262

[jsap13865-bib-0008] Faul, F. , Erdfelder, E. , Buchner, A. & Lang, A.G. (2009) Statistical power analyses using G*power 3.1: tests for correlation and regression analyses. Behavior Research Methods, 41, 1149–1160. 19897823 10.3758/BRM.41.4.1149

[jsap13865-bib-0009] Faul, F. , Erdfelder, E. , Lang, A.G. & Buchner, A. (2007) G*Power 3: a flexible statistical power analysis program for the social, behavioral, and biomedical sciences. Behavior Research Methods, 39, 175–191. 17695343 10.3758/bf03193146

[jsap13865-bib-0010] Ferreira‐Halder, C.V. , Faria, A.V.S. & Andrade, S.S. (2017) Action and function of Faecalibacterium prausnitzii in health and disease. Best Practice & Research. Clinical Gastroenterology, 31, 643–648. 29566907 10.1016/j.bpg.2017.09.011

[jsap13865-bib-0011] Ferrell, J.M. & Chiang, J.Y.L. (2019) Understanding bile acid signaling in diabetes: from pathophysiology to therapeutic targets. Diabetes and Metabolism Journal, 43, 257–272. 31210034 10.4093/dmj.2019.0043 PMC6581552

[jsap13865-bib-0012] Fracassi, F. , Pietra, M. , Boari, A. , Aste, G. , Giunti, M. & Famigli‐Bergamini, P. (2004) Breed distribution of canine diabetes mellitus in Italy. Veterinary Research Communications, 28, 339–342. 10.1023/b:verc.0000045441.77213.3b 15372992

[jsap13865-bib-0013] Gal, A. , Barko, P.C. , Biggs, P.J. , Gedye, K.R. , Midwinter, A.C. , Williams, D.A. et al. (2021) One dog's waste is another dog's wealth: a pilot study of fecal microbiota transplantation in dogs with acute hemorrhagic diarrhea syndrome. PLoS One, 16, e0250344. 33872339 10.1371/journal.pone.0250344 PMC8055013

[jsap13865-bib-0014] Galler, A.I. , Suchodolski, J.S. , Steiner, J.M. , Sung, C.H. , Hittmair, K.M. , Richter, B. et al. (2022) Microbial dysbiosis and fecal metabolomic perturbations in Yorkshire Terriers with chronic enteropathy. Scientific Reports, 12, 12977. 35902689 10.1038/s41598-022-17244-6 PMC9334271

[jsap13865-bib-0015] Guptill, L. , Glickman, L. & Glickman, N. (2003) Time trends and risk factors for diabetes mellitus in dogs: analysis of veterinary medical data base records (1970–1999). Veterinary Journal, 165, 240–247. 12672370 10.1016/s1090-0233(02)00242-3

[jsap13865-bib-0016] Han, X. , Wang, Y. , Zhang, P. , Zhu, M. , Li, L. , Mao, X. et al. (2021) Kazak faecal microbiota transplantation induces short‐chain fatty acids that promote glucagon‐like peptide‐1 secretion by regulating gut microbiota in db/db mice. Pharmaceutical Biology, 59, 1077–1087. 34392792 10.1080/13880209.2021.1954667 PMC8366640

[jsap13865-bib-0017] Iatcu, C.O. , Steen, A. & Covasa, M. (2021) Gut microbiota and complications of type‐2 diabetes. Nutrients, 14, 166. Available from: 10.3390/nu14010166 35011044 PMC8747253

[jsap13865-bib-0018] Ionut, V. , Castro, A.V. , Woolcott, O.O. , Stefanovski, D. , Iyer, M.S. , Broussard, J.L. et al. (2014) Hepatic portal vein denervation impairs oral glucose tolerance but not exenatide's effect on glycemia. American Journal of Physiology. Endocrinology and Metabolism, 307, E644–E652. 25117408 10.1152/ajpendo.00244.2014 PMC4200304

[jsap13865-bib-0019] Jergens, A.E. , Guard, B.C. , Redfern, A. , Rossi, G. , Mochel, J.P. , Pilla, R. et al. (2019) Microbiota‐related changes in unconjugated fecal bile acids are associated with naturally occurring, insulin‐dependent diabetes mellitus in dogs. Frontiers in Veterinary Science, 6, 199. 31316997 10.3389/fvets.2019.00199 PMC6610424

[jsap13865-bib-0020] Kang, A. , Kwak, M.J. , Lee, D.J. , Lee, J.J. , Kim, M.K. , Song, M. et al. (2024) Dietary supplementation with probiotics promotes weight loss by reshaping the gut microbiome and energy metabolism in obese dogs. Microbiology Spectrum, 12, e0255223. 38270436 10.1128/spectrum.02552-23 PMC10913549

[jsap13865-bib-0021] Laia, N.L. , Barko, P.C. , Sullivan, D.R. , McMichael, M.A. , Williams, D.A. & Reinhart, J.M. (2022) Longitudinal analysis of the rectal microbiome in dogs with diabetes mellitus after initiation of insulin therapy. PLoS One, 17, e0273792. 36067170 10.1371/journal.pone.0273792 PMC9447884

[jsap13865-bib-0022] Ma, G. , Pan, B. , Chen, Y. , Guo, C. , Zhao, M. , Zheng, L. et al. (2017) Trimethylamine N‐oxide in atherogenesis: impairing endothelial self‐repair capacity and enhancing monocyte adhesion. Bioscience Reports, 37, BSR20160244. 28153917 10.1042/BSR20160244 PMC5333780

[jsap13865-bib-0023] Mattin, M. , O'Neill, D. , Church, D. , McGreevy, P.D. , Thomson, P.C. & Brodbelt, D. (2014) An epidemiological study of diabetes mellitus in dogs attending first opinion practice in the UK. The Veterinary Record, 174, 349. 24570406 10.1136/vr.101950

[jsap13865-bib-0024] Minamoto, Y. , Minamoto, T. , Isaiah, A. , Sattasathuchana, P. , Buono, A. , Rangachari, V.R. et al. (2019) Fecal short‐chain fatty acid concentrations and dysbiosis in dogs with chronic enteropathy. Journal of Veterinary Internal Medicine, 33, 1608–1618. 31099928 10.1111/jvim.15520 PMC6639498

[jsap13865-bib-0025] Raimondi, F. , Santoro, P. , Barone, M.V. , Pappacoda, S. , Barretta, M.L. , Nanayakkara, M. et al. (2008) Bile acids modulate tight junction structure and barrier function of Caco‐2 monolayers via EGFR activation. American Journal of Physiology. Gastrointestinal and Liver Physiology, 294, G906–G913. 18239063 10.1152/ajpgi.00043.2007

[jsap13865-bib-0026] Rowlands, J. , Heng, J. , Newsholme, P. & Carlessi, R. (2018) Pleiotropic effects of GLP‐1 and analogs on cell signaling, metabolism, and function. Frontiers in Endocrinology, 9, 672. 30532733 10.3389/fendo.2018.00672 PMC6266510

[jsap13865-bib-0027] Salehi‐Sahlabadi, A. , Varkaneh, H.K. , Shahdadian, F. , Ghaedi, E. , Nouri, M. , Singh, A. et al. (2020) Effects of phytosterols supplementation on blood glucose, glycosylated hemoglobin (HbA1c) and insulin levels in humans: a systematic review and meta‐analysis of randomized controlled trials. Journal of Diabetes and Metabolic Disorders, 19, 625–632. 32550215 10.1007/s40200-020-00526-z PMC7270433

[jsap13865-bib-0028] Scott, S.A. , Fu, J. & Chang, P.V. (2020) Microbial tryptophan metabolites regulate gut barrier function via the aryl hydrocarbon receptor. Proceedings of the National Academy of Sciences of the United States of America, 117, 19376–19387. 32719140 10.1073/pnas.2000047117 PMC7431026

[jsap13865-bib-0029] Seldin, M.M. , Meng, Y. , Qi, H. , Zhu, W. , Wang, Z. , Hazen, S.L. et al. (2016) Trimethylamine N‐oxide promotes vascular inflammation through signaling of mitogen‐activated protein kinase and nuclear factor‐kappaB. Journal of the American Heart Association, 5, e002767. 26903003 10.1161/JAHA.115.002767 PMC4802459

[jsap13865-bib-0030] Tanase, D.M. , Gosav, E.M. , Neculae, E. , Costea, C.F. , Ciocoiu, M. , Hurjui, L.L. et al. (2020) Role of gut microbiota on onset and progression of microvascular complications of type 2 diabetes (T2DM). Nutrients, 12, 3719. Available from: 10.3390/nu12123719 33276482 PMC7760723

[jsap13865-bib-0031] Thaiss, C.A. , Levy, M. , Grosheva, I. , Zheng, D. , Soffer, E. , Blacher, E. et al. (2018) Hyperglycemia drives intestinal barrier dysfunction and risk for enteric infection. Science, 359, 1376–1383. 29519916 10.1126/science.aar3318

[jsap13865-bib-0032] Tilg, H. & Moschen, A.R. (2014) Microbiota and diabetes: an evolving relationship. Gut, 63, 1513–1521. 24833634 10.1136/gutjnl-2014-306928

[jsap13865-bib-0033] Wang, H. , Lu, Y. , Yan, Y. , Tian, S. , Zheng, D. , Leng, D. et al. (2019) Promising treatment for type 2 diabetes: fecal microbiota transplantation reverses insulin resistance and impaired islets. Frontiers in Cellular and Infection Microbiology, 9, 455. 32010641 10.3389/fcimb.2019.00455 PMC6979041

[jsap13865-bib-0034] Wang, Y. , Ye, X. , Ding, D. & Lu, Y. (2020) Characteristics of the intestinal flora in patients with peripheral neuropathy associated with type 2 diabetes. The Journal of International Medical Research, 48, 300060520936806. 32938282 10.1177/0300060520936806 PMC7503028

[jsap13865-bib-0035] Wiles, B.M. , Llewellyn‐Zaidi, A.M. , Evans, K.M. , O'Neill, D.G. & Lewis, T.W. (2017) Large‐scale survey to estimate the prevalence of disorders for 192 Kennel Club registered breeds. Canine Genetics and Epidemiology, 4, 8. 28932406 10.1186/s40575-017-0047-3 PMC5604186

[jsap13865-bib-0036] Winzeler, B. , da Conceicao, I. , Refardt, J. , Sailer, C.O. , Dutilh, G. & Christ‐Crain, M. (2020) Effects of glucagon‐like peptide‐1 receptor agonists on fluid intake in healthy volunteers. Endocrine, 70, 292–298. 32623637 10.1007/s12020-020-02394-2

[jsap13865-bib-0037] Wu, Z. , Zhang, B. , Chen, F. , Xia, R. , Zhu, D. , Chen, B. et al. (2022) Fecal microbiota transplantation reverses insulin resistance in type 2 diabetes: a randomized, controlled, prospective study. Frontiers in Cellular and Infection Microbiology, 12, 1089991. 36704100 10.3389/fcimb.2022.1089991 PMC9872724

[jsap13865-bib-0038] You, I. & Kim, M.J. (2021) Comparison of gut microbiota of 96 healthy dogs by individual traits: breed, age, and body condition score. Animals (Basel), 11, 2432. 34438891 10.3390/ani11082432 PMC8388711

[jsap13865-bib-0039] Zhang, P.P. , Li, L.L. , Han, X. , Li, Q.W. , Zhang, X.H. , Liu, J.J. et al. (2020) Fecal microbiota transplantation improves metabolism and gut microbiome composition in db/db mice. Acta Pharmacologica Sinica, 41, 678–685. 31937933 10.1038/s41401-019-0330-9 PMC7468362

[jsap13865-bib-0040] Zhou, Z. , Sun, B. , Yu, D. & Zhu, C. (2022) Gut microbiota: an important player in type 2 diabetes mellitus. Frontiers in Cellular and Infection Microbiology, 12, 834485. 35242721 10.3389/fcimb.2022.834485 PMC8886906

