# Supplementary figures and images for: The effect of lyophilised oral faecal microbial transplantation on functional outcomes in dogs with diabetes mellitus
Source: J Small Anim Pract. 2025 Apr 15;66(8):567–81. doi: 10.1111/jsap.13865 (PMC12331552; doi:10.1111/jsap.13865)

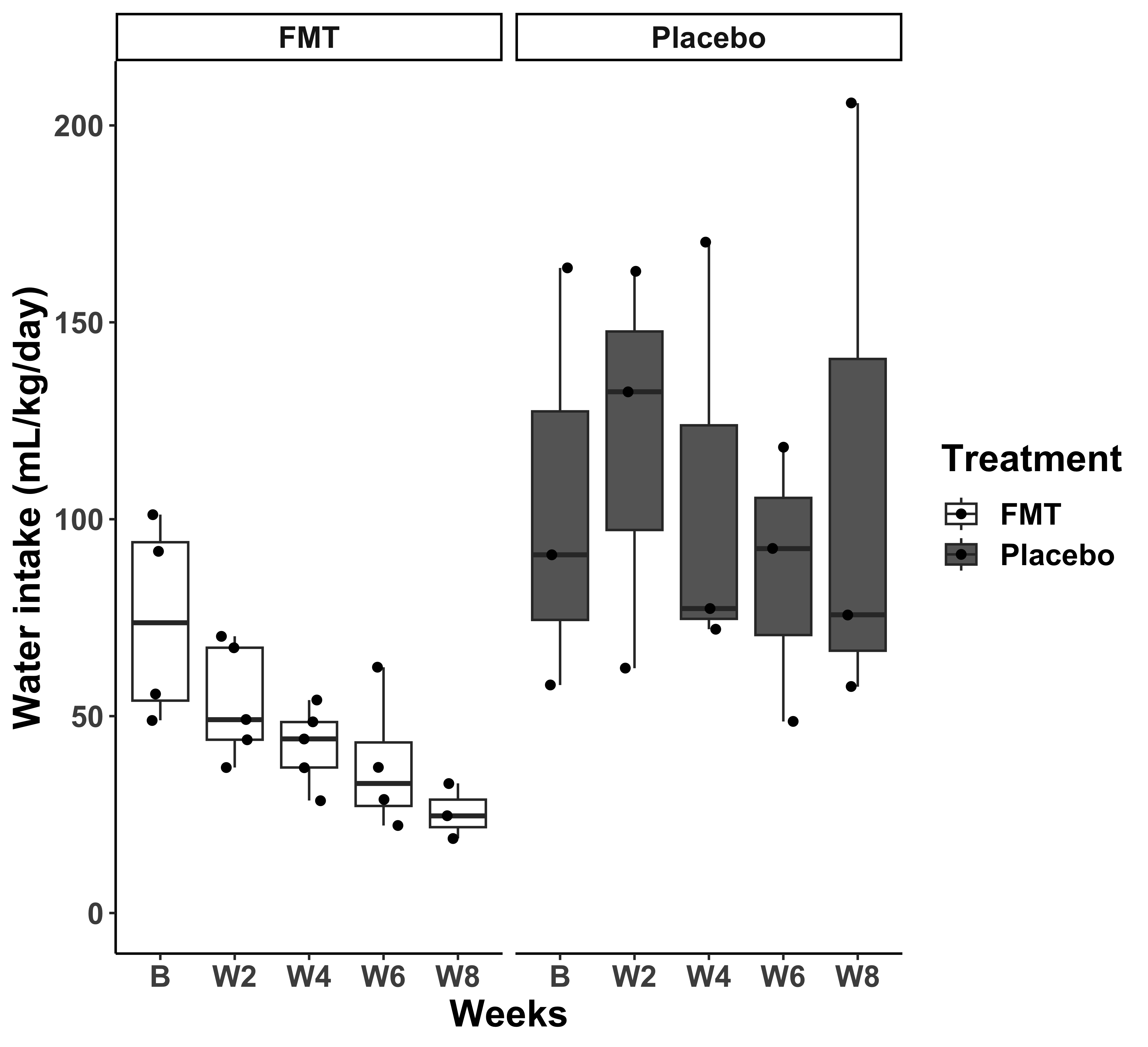

Supplement: Supplementary file 1 — Fig S1. 24‐hour water intake jitter boxplots. The box represents the 25th and 75th percentiles (IQR), the horizontal line represents the median, and the × represents the mean. The whiskers represent the maximum and minimum values below and above the upper (75th percentile + IQR) and lower (and 25th percentile − IQR) fences, respectively. Individual black dots represent individual dogs [file JSAP-66-567-s002.png]
