# Supplementary material for: The effect of lyophilised oral faecal microbial transplantation on functional outcomes in dogs with diabetes mellitus
Source: J Small Anim Pract. 2025 Apr 15;66(8):567–81. doi: 10.1111/jsap.13865 (PMC12331552; doi:10.1111/jsap.13865)
Supplement: Supplementary file 2 — Table S1. Dietary nutritional analysis of healthy and diabetic dog study participants [file JSAP-66-567-s001.docx]

| Patient | Commercial Diet | Total Daily Quantity | Guaranteed Analysis |
| --- | --- | --- | --- |
| Placebo dog 1 | Pedigree Tender Bites For Small Dogs - Chicken and Steak | 1/2 to 1 cup | Crude Protein 21.0%  Crude Fat 11.0%  Crude Fiber 4.0%  Moisture 12.0%  Linoleic Acid 2.0%  Calcium 1.0%  Phosphorus 0.8%  Zinc 80 mg/kg  Vitamin E 100 IU/kg  Glucosamine 350 mg/kg  Chondroitin Sulfate 275 mg/kg  335 Kcal/cup |
| Placebo dog 2 | Royal Canin Canine Satiety |  | Crude Protein 28.0%  Crude Fat 7.5 - 11.5%  Crude Fiber 14.8 - 18.8%  Moisture 10.0%  Glucosamine 1425 mg/kg  Chondroitin Sulfate 80 mg/kg  214 Kcal/cup |
| FMT dog 1 | Hill’s W/d (wet) | 1 - 13 oz can | Crude Protein 19.3%  Crude Fat 11.4%  Carbohydrates/NFE 50.4%  Crude Fiber 13.7%  Calcium 0.73%  Phosphorus 0.53%  Potassium 0.88%  Sodium 0.3%  Magnesium 0.105%  Vitamin C 187 ppm  Vitamin E 921 IU/kg  Total Omega-3 FA 1.12%  Total Omega-6 FA 2.82%  305 Kcal / 13 oz can |
| Placebo dog 3 | Hill’s W/d (dry) | 1 cup | Crude Protein 20.7%  Crude Fat 13.0%  Carbohydrates/NFE 45.2%  Crude Fiber 16.0%  Total Dietary Fiber 27.6%  Soluble Fiber 1.9%  Insoluble Fiber 25.5%  Calcium 0.8%  Phosphorus 0.66%  Potassium 0.78%  Sodium 0.27%  Magnesium 0.099%  Vitamin C 140 ppm  Vitamin E 682 IU/kg  Total Omega-3 FA 0.35%  Total Omega-6 FA 3.02%  Carnitine 515.8 ppm  255 Kcal/cup |
| FMT dog 2 | Taste of The Wild (dry) | 3 cups | Crude Protein 32.0%  Crude Fat 18.0%  Crude Fiber 3.0%  Moisture 10.0%  Calcium 1.2%  Phosphorus 1.0%  Zinc 150 mg/kg  Selenium 0.4 mg/kg  Vitamin A 10,000 IU/kg  DHA 0.05%  Taurine 0.12%  L-Carnitine 100 mg/kg  Vitamin E 150 IU/kg  Total Omega-3 FA 0.6%  Total Omega-6 FA 3.0%  Glucosamine 750 mg/kg  455 Kcal/cup |
| FMT dog 3 | Little Cesar Classic Loaf In Sauce | 4 - 3.5 oz containers | Crude Protein 9.0%  Crude Fat 4.0%  Crude Fiber 1.0%  Moisture 82.0%  90 Kcal/tray |
| FMT dog 4 | Hill’s Science Diet Sensitive Stomach and Skin (dry) | 1 and 1/3 cup | Crude Protein 25.0%  Crude Fat 16.8%  Carbohydrates/NFE 51%  Crude Fiber 1.4%  Calcium 0.95%  Phosphorus 0.78%  Potassium 0.87%  Sodium 0.35%  Magnesium 0.108%  Vitamin C 205 ppm  Vitamin E 674 IU/kg  Total Omega-3 FA 0.68%  Total Omega-6 FA 4.85%  Carnitine 22.8 ppm  Glucosamine 949 ppm  Chondroitin Sulfate 1499 ppm  394 Kcal/cup |
| Placebo dog 4 | Home cooked recipe  (beef, sweet potatoes, lentils, carrots) |  | Protein 39%  Fat 29%  Carbohydrates 24%  Fat-to-protein ratio 73% |
| FMT dog 5 | Rachel Ray Nutrish Salmon and Sweet Potato (dry) | 1/2 cup | Crude Protein 26.0%  Crude Fat 14.0%  Crude Fiber 5.0%  Moisture 10.0%  Calcium 1.2%  Phosphorus 1.0%  Zinc 250 mg/kg  DHA 0.1%  Taurine 0.2%  Vitamin C 55 mg/kg  Vitamin E 70 IU/kg  Total Omega-3 FA 1.0%  Total Omega-6 FA 2.0%  Glucosamine 400 mg/kg  Chondroitin Sulfate 200 mg/kg  355 Kcal/cup |
| Healthy Dog 1 | Purina Pro Plan Bright Mind 7+ | Unknown | Crude Protein 29.0%  Crude Fat 14.0%  Crude Fiber 3.0%  Moisture 12.0%  Arginine 1.2%  Linoleic Acid 1.7%  Eicosapentaenoic Acid (EPA) 0.15%  Docosahexaenoic Acid (DHA)0.15%  Calcium 1.0%  Phosphorus 0.8%  Selenium 0.35 mg/kg  Vitamin A 14,000 IU/kg  Vitamin E 500 IU/kg  Pyridoxine 3.0 mg/kg  Folic Acid 0.6 mg/kg  Ascorbic Acid (Vitamin C) 90 mg/kg  Glucosamine 500 ppm  Omega-3 Fatty Acids 0.45%  Omega-6 Fatty Acids 1.8%  423 kcal/cup |
| Healthy Dog 2 | Hill's Science Diet Adult 7+ dry mixed with Hill's Adult perfect weight (both chicken meal with barley recipes | Unknown | Crude Protein 18.7%  Crude Fat 13.7%  Carbohydrates/NFE 58.5%  Crude Fiber 2.4%  Calcium 1.16%  Phosphorus 0.68%  Potassium 0.92%  Sodium 0.32%  Magnesium 0.128%  Vitamin C 158 ppm  Vitamin E 598 IU/kg  DHA 0.002%  EPA 0.002%  Total Omega-3 FA 0.52%  Total Omega-6 FA 3.94%  Carnitine 324.9 ppm  Glucosamine 528 ppm  353 Kcal/cup |
| Healthy Dog 3 | Hill's Science Diet | Unknown | Crude Protein 23.9%  Crude Fat 14.7%  Carbohydrates/NFE 53.9%  Crude Fiber 2.1%  Calcium 0.75%  Phosphorus 0.56%  Potassium 0.76%  Sodium 0.3%  Magnesium 0.114%  Vitamin C 108 ppm  Vitamin E 524 IU/kg  Total Omega-3 FA 0.5%  Total Omega-6 FA 4.3%  363 Kcal/cup |
| Healthy Dog 4 | Kibble N'Bits (original) | Unknown | Crude Protein 19.0%  Crude Fat 8.0-12.0%  Crude Fiber 4.0%  Moisture 18.0%  Calcium 1.0%  Phosphorus 0.76%  Zinc 125 mg/kg  Vitamin A 5100 IU/kg  Vitamin D 500 IU/kg  375 Kcal/cup |
| Healthy Dog 5 | Purina One Chicken and Rice | Unknown | Crude Protein 26.0%  Crude Fat 16.0%  Crude Fiber 3.0%  Moisture 12.0%  Linoleic Acid 1.4%  Calcium 1.0%  Phosphorus 0.8%  Zinc 150 mg/kg  Selenium 0.35 mg/kg  Vitamin A 13,000 IU/kg  Vitamin E 250 IU/kg  Glucosamine 350 ppm  Omega-6 Fatty Acids 1.6%  360 kcal/cup |
| Healthy Dog 6 | Taste of The Wild | Unknown | Crude Protein 32.0%  Crude Fat 18.0%  Crude Fiber 3.0%  Moisture 10.0%  Calcium 1.2%  Phosphorus 1.0%  Zinc 150 mg/kg  Selenium 0.4 mg/kg  Vitamin A 10,000 IU/kg  DHA 0.05%  Taurine 0.12%  L-Carnitine 100 mg/kg  Vitamin E 150 IU/kg  Total Omega-3 FA 0.6%  Total Omega-6 FA 3.0%  Glucosamine 750 mg/kg  455 Kcal/cup |
| Healthy Dog 7 | Royal Canin Joint Diet | Unknown | Crude Protein 24.0%  Crude Fat 13.0%  Crude Fiber 7.6%  Moisture 10.0%  Chondroitin Sulfate 105mg/kg  314 Kcal/cup |
| Healthy Dog 8 | Diamonds Naturals Chicken Formula | Unknown | Crude Protein 26.0%  Crude Fat 16.0%  Crude Fiber 2.5%  Moisture 10.0%  DHA 0.05%  Zinc 150 mg/kg  Selenium (Se) 0.35 mg/kg  Vitamin A 10,000 IU/kg  Vitamin E 150 IU/kg  Omega-3 FA 2.5%  Omega-6 Fatty Acids 0.4%  421 kcal/cup |
| Healthy Dog 9 | Blue Buffalo, Chicken and Rice | Unknown | Crude Protein 24.0%  Crude Fat 14.0%  Crude Fiber 5.0%  Moisture 10.0%  Calcium 1.0%  Phosphorus 0.7%  Omega-3 FA 0.5%  Omega-6 FA 3.0%  Glucosamine 400 mg/kg  377 Kcal/cup |
